# Association of diet quality indices with serum and metabolic biomarkers in participants of the ORISCAV-LUX-2 study

**DOI:** 10.1007/s00394-023-03095-y

**Published:** 2023-03-14

**Authors:** Farhad Vahid, Axelle Hoge, James R. Hébert, Torsten Bohn, On behalf of the ORISCAV working group*., Ala’a Alkerwi, Stephanie Noppe, Charles Delagardelle, Jean Beissel, Anna Chioti, Saverio Stranges, Jean-Claude Schmit, Marie-Lise Lair, Marylène D’Incau, Jessica Pastore, Gloria Aguayo, Gwenaëlle Le Coroller, Michel Vaillant, Hanen Samouda, Brice Appenzeller, Laurent Malisoux, Sophie Couffignal, Manon Gantenbein, Yvan Devaux, Laetitia Huiart, Dritan Bejko, Guy Fagherazzi, Magali Perquin, Maria Ruiz-Castell, Isabelle Ernens

**Affiliations:** 1 Nutrition and Health Research Group, Department of Precision Health, Luxembourg Institute of Health, rue 1 A-B Thomas Edison, 1445 Strassen, Luxembourg; 2 Department of Public Health, University of Liège, Liège, Belgium; 3 South Carolina Statewide Cancer Prevention and Control Program and Department of Epidemiology and Biostatistics, Arnold School of Public Health, University of South Carolina, Columbia, SC USA; 4 Department of Nutrition, Connecting Health Innovations LLC (CHI), Columbia, SC USA

**Keywords:** Non-communicable diseases, Dietary patterns, Type 2 diabetes, Chronic disease risk, Oxidative stress, Inflammation, Systemic Immune-Inflammation Index (SII), Diet quality scores

## Abstract

**Purpose:**

Diet quality is a critical modifiable factor related to health, including the risk of cardiometabolic complications. Rather than assessing the intake of individual food items, it is more meaningful to examine overall dietary patterns. This study investigated the adherence to common dietary indices and their association with serum/metabolic parameters of disease risk.

**Methods:**

Dietary intakes of the general adult population (*n* = 1404, 25–79 years) were assessed by a validated food-frequency questionnaire (174 items). The French ANSES-Ciqual food composition database was used to compute nutrient intakes. Seven indicators were calculated to investigate participants’ diet quality: the Alternative Healthy Eating Index (AHEI), Dietary Approaches to Stop Hypertension Score (DASH-S), Mediterranean Diet Score (MDS), Diet Quality Index-International (DQI-I), Dietary Inflammatory Index (DII), Dietary Antioxidant Index (DAI), and Naturally Nutrient-Rich Score (NNRS). Various serum/metabolic parameters were used in the validity and association analyses, including markers of inflammation, blood glucose, and blood lipid status.

**Results:**

Following linear regression models adjusted for confounders, the DASH-S was significantly associated with most metabolic parameters (14, e.g., inversely with blood pressure, triglycerides, urinary sodium, uric acid, and positively with serum vitamin D), followed by the DQI-I (13, e.g., total cholesterol, apo-A/B, uric acid, and blood pressure) and the AHEI (11, e.g., apo-A, uric acid, serum vitamin D, diastolic blood pressure and vascular age).

**Conclusion:**

Food-group-based indices, including DASH-S, DQI-I, and AHEI, were good predictors for serum/metabolic parameters, while nutrient-based indices, such as the DAI or NNRS, were less related to biological markers and, thus, less suitable to reflect diet quality in a general population.

**Supplementary Information:**

The online version contains supplementary material available at 10.1007/s00394-023-03095-y.

## Introduction

The quality of the diet, along with other lifestyle factors, such as physical activity and smoking, which are regarded as being among the most critical modifiable factors related to the incidence of several non-communicable diseases (NCDs) and health issues, including cardiovascular disease (CVD) [1], type 2 diabetes (T2D), obesity/overweight [2, 3], some types of cancer [4] and nonalcoholic fatty liver disease (NAFLD) [5]. Many of these NCDs are surging, e.g., the age-standardized prevalence of T2D in adults has almost doubled, from 4.7% in 1980 to 8.5% in 2014, though heterogeneity in the global distribution is considerable [6].

Despite that much emphasis has been placed on individual dietary constituents, such as limiting sugar and salt, as well as saturated fat intake or increasing dietary fiber consumption, it has been highlighted that the overall dietary patterns constitute a better marker for the healthiness of a diet [7]. It is possibly better suited to be related to the risk of certain chronic diseases than individual food items alone [8]. Indeed, studies have shown that diet quality can be considered as an independent factor for predicting the risk of various diseases [2–5].

However, it is paramount to define the best scheme or method to evaluate the overall quality of the diet in different populations. For this purpose, several indices have been developed that capture various aspects of the diet. Though these dietary indices partly focus on different aspects of the diet, almost all of them strive to provide a comprehensive and complete perspective of dietary quality regarding a specific target, such as the intake of antioxidants. In contrast to nutritional surveys investigating only macro-micronutrient intake, the indices aim to examine various aspects of a person's diet, such as variety, balance, adequacy, and health-related aspects [9], considering the intake of certain nutrients (nutrient-based indices) and/or food items (food-group-based indices). For example, the Healthy Eating Index (HEI), a food-group-based index, has been designed to examine the overall adherence of individuals to the 2015–2020 Dietary Guidelines for Americans [10]. A recent study showed that the HEI-2015 and its constituents was associated with inflammatory biomarkers, e.g., lower circulating c-reactive protein (CRP) and interleukin-6 (IL-6) concentrations, as well as white blood cell (WBC) counts [11]. Likewise, an exploratory analysis concluded that there existed a significant association between the HEI and total serum antioxidant capacity and inflammatory markers, including tumour necrosis factor α (TFN-α) and Il-6 [12]. As another example, the Diet Quality Index-International (DQI-I), a nutrient and food-group-based index, is one of the indices designed to assess the overall quality of an individual's diet [13]. The DQI-I includes four scoring subgroups that examine complementary aspects of diet, i.e., variety, moderation, adequacy, and overall diet balance (Supplementary Tables 1 and 4) [13, 14]. Studies have shown that the DQI-I correlated with several biomarkers associated with cardiometabolic risk factors, including inversely with total serum cholesterol, body mass index (BMI), and positively with high-density lipoprotein cholesterol (HDL-c), among others [14–16].

Some indices focus on more specific aspects of diet-related disease risk. For example, the Dietary Inflammatory Index (DII) focuses mainly on the pro- vs. anti-inflammatory properties of the diet, as many NCDs have been related to chronic inflammation, such as CVD [1], diabetes [17], cancer [18], NAFLD [19], and obesity [20]. The positive association of the DII with inflammation-related biomarkers such as TNF-α, IL-6, and CRP and more indirect biomarkers such as the serum levels of insulin and the erythrocyte sedimentation rate (ESR) has been shown in several studies [1, 17–19]. The Dietary Antioxidant Index (DAI) is another example where the main focus rests on the antioxidant properties of the diet [21], as increased oxidative stress levels also characterize many NCDs.

Nonetheless, each of these indices has inherent strengths and limitations, and some inevitably overlap, despite showing complementarity. For instance, strong correlations have been found between the DQI-I and Dietary Approaches to Stop Hypertension Score (DASH-S) [22]. Moreover, a systematic review and meta-analysis of cohort studies concluded that dietary indices such as DASH-S were associated with health status, including all-cause mortality, CVD and cancer incidence or mortality, T2D, and neurodegenerative disease and related (bio)markers, including inflammatory indicators and body composition, among others [23]. However, due to the large number of published dietary indices and, in part, their large diversity, choosing an index that can thoroughly analyze dietary quality and correlate with the targeted health outcome, such as specific biological endpoints, is challenging. Systematic and narrative reviews have attempted to identify/introduce the most suitable and effective index to capture total dietary patterns; however, this is impossible due to the complexity of the diet and its many food components and eating habits/patterns. According to conclusions stated in several reviews, rather than pursuing a “one size fits all”, the best strategy may be to choose the most appropriate index or indices depending on the research question, taking into account the strengths and limitations of that index/those indices.

In the present study, we selected a range of frequently used indices (nutrient-based, food-group-based, as well as food-group and nutrient-based indices) to examine the association between the quality of the diet and selected biomarkers of disease risk in a general adult population residing in Luxembourg, taking part in the second wave of the ORISCAV-LUX study. The indices were calculated based on valid food-frequency questionnaires (FFQs) and were associated with selected serum and metabolic parameters.

## Materials and methods

### Study population and design

The full study protocol and method description have been published previously [24, 25]. Briefly, the findings are based on the second wave of the Observation of Cardiovascular Risk Factors in Luxembourg (ORISCAV-LUX 2; 2016–2017), the second nationwide study on CVD prevalence and related risk factors in the Luxembourgish adult population. This is a follow-up to the ORISCAV-LUX 1 study, which was implemented on adults residing in Luxembourg aged 18–69 years in 2007–2008 [25]. Luxembourgish residents aged 25–81 years, with a total of 1558 persons, were enrolled in the second wave, ORISCAV-LUX 2. As the research protocol stipulated that people until 79 years of age can participate, one participant (81 years old) was excluded from the analyses. In addition, participants without anthropometric and energy intake data (*n* = 7), FFQ (*n* = 120), and extreme values (top 1 and bottom percentile) in dietary energy intakes (*n* = 26) were excluded from the final analyses. Therefore, 1404 individuals were retained, i.e., they delivered a complete dataset including the nutritional aspects.

### General data collection

Data from questionnaires related to lifestyle, sociodemographic aspects, and self-reported health conditions were included. Clinical measurements and anthropometrics were also assessed, as well as scheduled appointments at a private accredited laboratory (Ketterthill, Esch-sur-Alzette, Luxembourg) for blood and urine sample collections and analyses. All participants were informed about the objectives of the study orally and in written and consented to participate in the survey (written consent was obtained from all participants). The study was approved by the National Research Ethics Committee (CNER, No. 201-505/12) and the National Commission for Data Protection (CNPD).

Data on age, gender, education, job, income, and marital status were collected using a general information questionnaire. A trained nurse carried out the anthropometric measures, including weight, height, and waist circumference (WC). The body weight (kg), height (cm), and WC (cm) were measured in a light dress without shoes using a digital scale. The participants’ BMI was assessed as weight (kg) divided by the square of height in meters (kg/m^2^).

### Assessment of dietary intakes and indices scoring algorithms

The individuals completed a validated quantitative food-frequency questionnaires (FFQ) [26] under the supervision of a nurse. The frequency and quantity of 174 food and beverage items were documented to assess dietary intakes. A frequency ranging from ‘never/rarely’, ‘one–three times/month’, ‘one–two times/week’, ‘three–five times/week’ ‘once a day’, to ‘twice or more a day’, and portion size images were used to estimate macro-and micronutrient intakes. The daily food and nutrient intakes were calculated by multiplying the frequency of consumption by the portion sizes of all food items and considering the content of macro-or micronutrients as listed in the French ANSES-Ciqual food composition database (indexing the nutritional composition of > 3100 food items) [27]. The results were employed to determine the selected dietary indices (Table 1). The full description of the calculation of the indices and their scoring algorithm is provided in the supplementary file (Supplementary Tables 1–5); however, we briefly describe them here:

**Table 1:** The dietary indices investigated in the present study, along with their short descriptions/cut-off values

Index	Abbreviation	Basis	Index components	Cut-off values/scoring	Scoring range	References
Alternative Healthy Eating Index	AHEI	Dietary Guidelines for Americans, Food Guide Pyramid	Vegetables; fruit; nuts and soy; a ratio of white to red meat; fiber; a ratio of PUFA to SFA; multivitamin use; alcohol (male/female)	Total score: 0 (poor diet) to 75 (excellent diet). Energy intake was not considered, and all items get equal weight, except for multivitamin use (the multivitamin component is dichotomous, contributing either 5 points (consuming any supplement) or zero (for all others) to avoid over-weighting this component)	0–75	[29]
Mediterranean Diet Score	MDS	Specific dietary pattern: Mediterranean dietary pattern	MUFA to SFA ratio; legumes; grains; fruits and nuts; vegetables; meat and meat products; milk and dairy products; alcohol	The sample's median was used as a cut-off point, dichotomous and population-specific. Total score: 0 (poor diet) to 9 (excellent diet);—equal weight for all items	0–9	[30]
Dietary Approaches to Stop Hypertension Score	DASH-S	Dietary approaches to stop hypertension recommendations	Fruits; vegetables; nuts and legumes; dairy products; whole grains; sodium; sweetened beverages; red, processed meats	Cut-off points in servings per day for low and high consumption were based on quintiles of intake; all components are equally weighted	8–40	[32]
Diet Quality Index-International	DQI-I	Worldwide and individual national dietary guidelines, the Food Guide Pyramid	Overall food group variety; within-group variety; vegetables; fruits; grains; fiber; protein; iron; calcium; vitamin C; total fat; SFA; cholesterol; sodium; empty energy foods; macronutrient ratio; FA ratio	Total score: 0 (poor diet) to 100 (excellent diet). Three levels of energy intake were used for the recommended intake of fruits, vegetables, grains, and fiber—different weights for different items	0–100	[13]
Dietary Inflammatory Index	DII	Literature-derived, population-based scoring algorithm	Forty-five food and nutrient parameters having an impact on inflammatory biomarkers	Its respective ‘overall food parameter-specific inflammatory effect score’ to obtain the ‘food parameter-specific DII score’ multiplies the centered percentile value for each food parameter. Afterward, the ‘food parameter-specific DII scores’ were summed up to create an individual’s overall DII score	Typical published range: − 8.88 to + 8.00	[34]
Dietary Antioxidant Index	DAI	Population-based scoring algorithm	Six antioxidant vitamins and minerals (vitamins A, C, E, and selenium, magnesium, and zinc)	The DAI was calculated by summing up the standardized intake of these vitamins and minerals with equal weight	N/A	[35]
Naturally Nutrient-Rich Score	NNRS	Mean daily percentage values (DVs) for 14 nutrients per 2000 kcal food	Protein; calcium; iron; vitamin A; vitamin C; thiamine; riboflavin; vitamin B-12; folate; vitamin D; vitamin E; MUFA; potassium; zinc	The NNRS was based on a nutrient-to-calorie ratio. The NNRS is the average of DVs for 14 key nutrients	%	[40]

All references are the first study in which the index was used/fully described

*PUFA* polyunsaturated fatty acids, *SFA* saturated fatty acids, *MUFA* monounsaturated fatty acids, *FA* fatty acids

#### Alternate Healthy Eating Index (AHEI)

The AHEI was developed as an alternative to the Healthy Eating Index (HEI). It is based on foods that may prevent chronic disease risk and comprises 13 components that entail different food groups and recommendations [28]. The AHEI-2010 constitutes an updated version and shows more advantages than the HEI for predicting major chronic disease and CVD risks [10, 29]. All individual component scores were summed up for a total AHEI score ranging from 0 (worst) to 75 (best) (Supplementary Table 2).

#### Mediterranean Diet Score (MDS)

Another frequently applied index is the Mediterranean Diet Score (MDS), which measures adherence to the Mediterranean diet (MD) [30, 31]. The MD is one of the most well-known diets related to reducing the risk of CVD and other related diseases. Using the population-specific medians among the participants as cut-off values, points of 0 or 1 were assigned to each of the 9 indicated items. This MDS can, thus, take a score from 0 points (minimal adherence) to 9 (maximal adherence).

#### Dietary Approaches to Stop Hypertension Score (DASH-S)

DASH-S [32] measures how people adhere to a diet that is related to a lower risk of hypertension (DASH), though associated outcomes such as CVD and diabetes have also been examined [33]. This index's main feature is considering individuals' sodium intake, which generally remains above recommendations in Western cultures. We classified participants into quintiles for each component according to their intake ranking. We then summed up the component scores to attain an overall DASH-S ranging from 8 to 40 (Supplementary Table 3).

#### Diet Quality Index-International (DQI-I)

Based on dietary guidelines, the DQI-I is designed and developed based on international recommendations by the FAO/WHO [13, 14]. This index comprehensively integrates different aspects of the diet and examines public health nutrition in various communities [13, 14]. The four major categories (Supplementary Table 4) are variety, adequacy, moderation, and overall balance of the diet—with total scores ranging from 0 (poorest diet) to 100 (highest possible score, excellent diet).

#### Dietary Inflammatory Index (DII®)

The DII aims to study diet-induced inflammation [34] and includes 45 food items (anti-inflammatory ones such as dietary fiber and pro-inflammatory ones such as red meat). The DII has been validated in several human studies by CRP, TNF-α, IL-6, and other inflammatory biomarkers, and thus can predict, to some extent, the serum levels of these biomarkers in relation to diet and has been correlated with a large number of NCDs [18, 34]. The computation of the DII is based on dietary intake data linked to the regionally representative world database that provides an accurate and robust assessment of each parameter's mean and standard deviation [34]. These then become the multipliers to represent an individual's exposure relative to the 'standard global mean' as a Z-score. This is attained by subtracting the 'standard mean' from the reported amount and dividing this value by its standard deviation (means and standard deviations for all 45 parameters are shown in Supplementary Table 5). From those 45 parameters, in our study, there were 32 available items to calculate the DII. According to validation reports, using even only 21 out of 45 items can correctly predict serum inflammatory biomarkers [35].

#### Dietary Antioxidant Index (DAI)

The DAI focuses on antioxidant diets. Since the Western diet (a high-fat, refined-carbohydrate diet) has often been associated with a pro-oxidant/antioxidant imbalance [36], a diet fostering antioxidant reactions that counteract the effects of reactive oxygen species (ROS) can contribute to the prevention or treatment of oxidative stress-related diseases [37, 38]. Of note, there is a close relationship between oxidative stress and inflammation [39]. By standardizing the intake of six major dietary antioxidants, including vitamins A, E, and C, and magnesium, zinc, and selenium (the minerals participating in enzymatic antioxidant reactions), the DAI can predict the antioxidant properties of the diet and thus, the risk of various disease outcomes such as cancer [21], obesity [38] and CVD [37]. The DAI has been validated using biological measures, including total antioxidant capacity (TAC) and malondialdehyde (MDA) in plasma/serum [21].

$${\text{DAI}} = \sum\limits_{{i = 1}}^{{n = 6}} {\frac{{{\text{Individual intake}} - {\text{Global mean}}}}{{{\text{Global SD}}}}/n}$$

*n* = the number of antioxidants included in the formula; *i* = this formula is calculated separately for each antioxidant and finally divided by *n*; Global means and SDs = are extracted from the reference database.

#### Naturally Nutrient-Rich Score (NNRS)

The Naturally Nutrient-Rich Score (NNRS) is based on a nutrient-to-calorie ratio [40]. This index is one of the few indices that examines the quantity of micronutrients based on guidelines, e.g., a report of a Joint FAO/WHO consultation, and its primary purpose is to ensure adequate intake of micronutrients to improve the quality of diet. Fourteen essential key nutrients and recommended daily values (DVs) for each 2000 kcal energy intake based on the USA dietary reference intakes (DRI) were used to calculate the NNRS (Table 1 and Supplementary Table 1).

$${\text{NNRS}} = \sum \% {\text{DV 2}}000 {\text{ kcal}}/{14}{\text{}}$$

### Assessment of physical activity

A short form of the International Physical Activity Questionnaire (IPAQ) was used to estimate physical activity [41]. This IPAQ is a self-reported validated 7-item measure of physical activity over the past week. The amount of time that each individual spent on an activity was multiplied by the corresponding metabolic equivalent of task (METs) while considering the frequency of engagement during the past seven days. The continuous score of physical activity, expressed as METs-min per week, was then obtained by summing up the scores for the different activities (walking, moderate-intensity, and vigorous-intensity activities).

### Measurement of blood/urine parameters

After overnight fasting, venous blood samples were drawn, and urine samples were collected as early morning midstream urine specimens. All blood and urine samples were stored in the Integrated BioBank of Luxembourg (IBBL), and a commercial accredited company (Ketterthill) later performed the analyses. From the blood samples, we obtained fasting blood glucose (FBG), high sensitive C-reactive protein (hs-CRP), apo-A and B, triglycerides, total cholesterol, low-density lipoprotein cholesterol (LDL-c), high-density lipoprotein cholesterol (HDL-c), free triiodothyronine (FT3) and free thyroxine (FT4) hormones, thyroid-stimulating hormone (TSH), insulin, and glycated hemoglobin (HbA1C), hematocrit, and hemoglobin, as well as serum levels of sodium, uric acid, creatinine, magnesium, potassium, calcium, magnesium, ferritin, and 25(OH) vitamin D. From the spot urine samples, we measured microalbuminuria, creatinine, and urinary sodium concentration. In addition, using FBG and insulin levels, we estimated the Homeostatic Model Assessment for Insulin Resistance (HOMA-IR).

### Systemic Immune-Inflammation Index (SII)

The SII is a promising prognostic indicator for systemic immune-inflammation-related conditions [42]. In fact, the SII assesses three of the homeostatic system markers that play a role in the inflammatory procedure: platelets, lymphocytes, and neutrophils. This index correlated with low-grade inflammation, characterized by a mildly elevated CRP [43]. Similar to increased serum levels of CRP, the evidence indicates that platelet/lymphocytes/neutrophils parameters are biomarkers that reflect a systemic inflammatory response [43].

The SII was estimated as total platelet count (*P*) × neutrophil-to-lymphocyte ratio (*N*/*L*) [44].

$${\text{SII}} = P \times N/L\;{\text{ratio}}{}$$

### Vascular and kidney function

A trained and experienced nurse measured systolic and diastolic blood pressure (SBP and DBP) several times (a standardized method was applied). The average (mean) of the measurements was used as the final variable. In addition, arterial age was determined as the average age for a given carotid-femoral pulse wave velocity (PWV). PWV, central systolic and diastolic blood pressure, arterial age, and blood pressure in a lying position were measured with Complior™. The PWV was estimated by dividing the carotid-femoral distance by the transit time of the forward-traveling pulse between the carotid and femoral arteries.

In addition, glomerular filtration rate (GFR) as a vascular function-related measurement was estimated by the Modification of Diet in Renal Disease (MDRD) method. The MDRD was evaluated using an equation based on six variables: age, gender, ethnicity, serum creatinine, urea, and albumin [45].

### Statistical analysis

The normality of the data distribution and homogeneity of variance was assessed using Q–Q normality plots and the Kolmogorov–Smirnov test (KS test), and a box plot. For the non-normally distributed data, a log-transformation was performed. For a first explorative purpose, bivariate correlation analyses with Spearman-rank correlation coefficients were calculated.

To study the association between the dietary indices and serum and all metabolic parameters, linear regression modeling in SPSS was carried out. This included a set of confounders that were chosen due to physiological plausibility and based on literature. For models, two-sided p-values above 0.1 were selected as means for elimination. This step resulted in acquiring a model (saturated model) from the thorough batch of variables by automatically (step-down procedure) dismissing those that did not contribute significantly to the model. The respective dietary quality indices were the explanatory, independent variable, while the measured metabolic parameters were the observed, dependent outcome variable.

## Results

### General characteristics of the population

The distribution of participants' characteristics in the quartiles of dietary indices is presented in Table 2. Table 2 shows the level of adherence of the participants in the sociodemographic groups to different indicators. For example, older people (> 65 years) were more adherent to the AHEI or MDS (being in a higher index score quartile) than younger people (≤ 34.99 years). In addition, the distribution (median, interquartile range) of participants' biomarkers and diet quality indices according to age and gender groups are presented in Table 3.

**Table 2:** Distribution of participants' characteristics taking part in the ORISCAV-LUX 2 study, according to the quartiles of the investigated dietary indices (*n* = 1404 participants)

Characteristics	AHEI^1^				MDS^2^				DASH-S^3^				DQI-I^4^				DII^5^				DAI^6^				NNRS^7^			
	Q1	Q2	Q3	Q4	Q1	Q2	Q3	Q4	Q1	Q2	Q3	Q4	Q1	Q2	Q3	Q4	Q1	Q2	Q3	Q4	Q1	Q2	Q3	Q4	Q1	Q2	Q3	Q4
Age categories (y)																												
≤ 35 (*n* = 162, 11.5%)	54	40	31	37	54	34	35	39	56	44	33	29	53	36	42	31	40	34	46	42	40	46	41	35	41	44	35	42
35–44.99 (*n* = 315, 22.4%)	84	97	70	64	102	75	68	70	110	87	77	41	105	73	76	61	75	84	70	86	77	73	82	83	74	79	81	81
45–54.99 (*n* = 381, 27.1%)	93	101	88	99	107	102	82	90	108	96	91	86	98	103	85	95	92	110	90	89	90	92	102	97	89	94	95	103
55–64.99 (*n* = 352, 25.1%)	76	97	79	100	100	74	74	104	80	83	92	97	80	77	90	105	99	80	92	81	90	91	79	92	92	92	86	82
> 65 (*n* = 194, 13.8%)	42	55	40	57	52	54	36	52	43	41	56	55	47	38	47	62	43	44	54	53	56	49	47	42	56	46	53	39
Gender																												
Men (*n* = 654, 46.6%)	172	184	143	155	222	169	141	122	242	162	155	95	224	155	137	138	186	170	153	145	129	132	176	217	120	139	177	218
Women (*n* = 750, 53.4%)	177	206	165	202	193	170	154	233	155	189	192	214	159	172	203	216	163	182	199	206	224	219	175	132	232	216	173	129
Education level																												
No diploma* (*n* = 182, 13.0%)	49	45	41	47	40	45	41	56	54	40	39	49	50	36	31	66	33	50	44	55	42	55	38	47	42	48	44	48
Certificated** (*n* = 251, 17.9%)	74	74	60	43	94	68	41	48	89	69	44	49	79	60	55	57	50	64	60	77	72	54	65	60	71	66	54	60
Diploma*** (*n* = 321, 22.9%)	88	81	65	87	106	71	64	80	89	86	89	57	89	81	76	75	86	75	79	81	84	70	88	79	85	69	90	77
Tertiary**** (*n* = 526, 37.5%)	110	144	117	155	138	128	126	134	130	130	138	128	136	120	142	128	156	133	130	107	118	139	131	138	114	144	130	138
Did not answer (*n* = 124, 8.8%)	28	46	25	25	37	27	23	37	35	26	37	26	29	30	36	29	24	30	39	31	37	33	29	25	40	28	32	24
Smoking status																												
Non-smoker (*n* = 1218, 86.8%)	287	340	271	320	349	287	260	322	317	307	309	285	305	285	297	331	302	308	311	297	308	310	306	294	305	320	306	287
BMI categories (kg/m^2^)																												
≤ 25 (*n* = 649, 46.2%)	148	161	144	196	174	140	148	187	150	153	167	179	148	136	182	183	159	177	157	156	175	168	166	140	172	180	159	138
25–29.99 (*n* = 493, 35.1%)	126	157	102	108	151	135	102	105	157	132	119	85	150	132	102	109	124	114	122	133	119	130	121	123	118	123	131	121
≥ 30 (*n* = 262, 18.7%)	75	72	62	53	90	64	45	63	90	66	61	45	85	59	56	62	66	61	73	62	59	53	64	86	62	52	60	88
WHR																												
> 0.85 in women (*n* = 305, 40.6%)	65	79	78	83	74	72	68	91	68	72	78	87	57	75	76	97	71	77	83	74	76	87	78	64	85	76	78	66
> 0.90 in men (*n* = 470, 71.8%)	125	146	99	100	165	126	95	84	178	120	106	66	165	120	86	99	127	119	115	109	96	95	128	151	94	96	124	156
Marital status																												
Single^a^ (*n* = 36, 2.6%)	7	8	8	13	10	6	5	15	2	8	12	14	3	8	7	18	9	7	10	10	13	11	3	9	14	11	2	9
Married (*n* = 1047, 74.6%)	254	298	236	259	304	259	222	262	293	270	251	232	288	250	251	258	261	268	268	250	254	266	267	260	245	275	273	254
Widow(er) (*n* = 165, 11.7%)	51	38	26	49	58	33	37	36	62	37	35	30	53	34	43	34	46	31	43	44	42	38	41	43	45	34	35	50
Divorced^b^ (*n* = 156, 11.1%)	37	44	38	36	42	41	31	41	40	36	48	31	39	34	38	44	32	46	31	46	43	36	40	36	47	35	40	33
Occupation (job)																												
Employed (*n* = 918, 65.4%)	246	247	207	218	281	227	194	216	291	233	221	173	269	226	224	199	229	234	225	230	218	234	232	234	219	231	230	238
Unemployed^c^ (*n* = 153, 10.9%)	29	39	36	49	45	25	28	55	26	42	40	45	31	31	39	52	41	42	34	36	40	36	39	38	40	42	35	36
Retired, leave^d^ (*n* = 316, 22.5%)	74	95	62	85	84	87	70	75	78	74	81	83	81	67	72	96	78	69	86	83	90	76	76	74	88	77	83	68
Did not answer (*n* = 17, 1.2%)	0	9	3	5	5	0	3	9	2	2	5	8	2	3	5	7	1	7	7	2	5	5	4	3	5	5	2	5
Country of birth																												
Luxembourg (*n* = 832, 59.3%)	200	233	188	211	255	207	165	205	233	206	207	186	229	209	191	203	218	202	197	215	214	201	205	212	215	202	203	212
Portugal (*n* = 110, 7.8%)	28	38	20	24	33	29	27	21	37	33	22	18	29	27	32	22	21	26	39	24	23	35	29	23	26	29	28	27
Other European countries (*n* = 336, 23.9%)	90	89	73	84	91	80	72	93	103	81	87	65	100	66	79	91	79	86	87	84	84	89	83	80	83	91	85	77
Non-European countries (*n* = 126, 9.0%)	31	30	27	38	36	23	31	36	24	31	31	40	25	25	38	38	31	38	29	28	32	26	34	34	28	33	34	31
Income (EUR/month)																												
Less than 750 (*n* = 4, 0.3%)	1	1	0	2	1	0	0	3	1	1	0	2	1	0	1	2	2	1	1	0	0	0	2	2	0	0	1	3
750–1499 (*n* = 22, 1.6%)	7	5	5	5	5	3	6	8	5	6	7	4	4	4	8	6	7	2	6	7	8	3	4	7	6	6	2	8
1500–2249 (*n* = 49, 3.5%)	9	11	12	17	11	11	11	16	8	10	18	13	7	9	13	20	12	15	10	12	10	9	14	16	11	9	11	18
2250–2999 (*n* = 78, 5.6%)	19	20	19	20	19	17	12	30	20	18	14	26	19	10	17	32	19	14	21	24	22	16	19	21	26	18	13	21
3000–4999 (*n* = 335, 23.9%)	91	82	83	79	111	77	70	77	102	84	91	58	99	76	79	81	77	85	87	86	80	84	84	87	82	80	86	87
5000–10,000 (*n* = 482, 34.3%)	129	135	93	125	145	123	112	102	141	132	118	91	148	112	111	111	112	137	119	114	123	127	118	114	118	136	114	114
More than 10,000 (*n* = 115, 8.2%)	18	34	28	35	26	26	34	29	28	25	31	31	27	34	33	21	45	31	25	14	18	29	31	37	17	27	36	35
Did not answer (*n* = 319, 22.7%)	75	102	68	74	97	97	50	90	92	75	68	84	78	82	78	81	75	67	83	94	92	83	79	65	92	79	87	61

*AHEI* Alternative Healthy Eating Index, *MDS* Mediterranean Diet Score, *DASH-S* Dietary Approaches to Stop Hypertension Score, *DQI-I* Diet Quality Index-International, *DII* Dietary Inflammatory Index, *DAI* Dietary Antioxidant Index, *NNRS* Naturally Nutrient-Rich Score, *Q* Quartile, *WHR* Waist–hip ratio

*Pre-primary and primary education

**CATP—Certificate of Technical and Professional Aptitude, CITP—Certificate of Technical and Professional Initiation, CCM—Certificate of Manual Capability, Diploma for Completion of Secondary Technical Studies, Diploma for Completion of Secondary General Studies

***Technician diploma, Bac + 2 (BTS), Bac + 3 (Bachelors/Degree), Diploma from a Grande Ecole, an Engineering School

****Bac + 4 (Masters), Bac + 5 and more (3rd Cycle, DEA, DESS, MBA, Masters, Ph.D., etc.)

^a^Single, never married, and never in a registered partnership

^b^Divorced, separated, separated but still legally married

^c^In school, university or training, at home, unemployed or in search of employment

^d^Retired or in early retirement, on long-term leave

^1^Q1 (*n* = 349, 24.9%); Q2 (*n* = 390, 27.8%); Q3 (*n* = 308, 21.9%); Q4 (*n* = 357, 25.4%)

^2^Q1 (*n* = 415, 29.6%); Q2 (*n* = 339, 24.1%); Q3 (*n* = 295, 21.0%); Q4 (*n* = 355, 25.3%)

^3^Q1 (*n* = 397, 28.3%); Q2 (*n* = 351, 25.0%); Q3 (*n* = 347, 24.7%); Q4 (*n* = 309, 22.0%)

^4^Q1 (*n* = 383, 27.3%); Q2 (*n* = 327, 23.3%); Q3 (*n* = 340, 24.2%); Q4 (*n* = 354, 25.2%)

^5, 6, and 7^Q1 (*n* = 349, 24.9%); Q2 (*n* = 352, 25.1%); Q3 (*n* = 352, 25.1%); Q4 (*n* = 351, 25.0%)

**Table 3:** Median (interquartile range) of participants' biomarkers and diet quality indices according to age and gender groups (n = 1404 participants)

	Age groups					Gender		Total
	≤ 35	35–44.99	45–54.99	55–64.99	> 65	Men	Women	
AHEI	32 (18)	33 (16)	35 (17)	36 (17)	35 (18)	35 (17)	35 (17)	35 (17)
MDS	4 (2)	4 (2)	4 (2)	5 (3)	4 (3)	4 (2)	5 (3)	4 (3)
DASH-S	23 (6)	23 (6)	24 (6)	25 (6)	25 (6)	23 (6)	25 (6)	24 (6)
DQI-I	63.5 (12)	64 (11)	64 (11)	65.5 (11)	66 (12)	63 (11)	66 (10)	64 (11)
DII	− 1.75 (2.79)	− 2.04 (2.91)	− 2.12 (2.60)	− 2.11 (2.74)	− 1.82 (2.78)	− 2.22 (2.71)	− 1.88 (2.98)	− 2.02 (2.70)
DAI	2.47 (7.07)	3.15 (8.40)	3.43 (7.73)	2.67 (8.30)	2.15 (6.78)	4.16 (8.52)	1.75 (6.39)	2.86 (7.94)
NNRS	126.4 (61.5)	131.88 (59.7)	130.5 (62.4)	125.7 (59.8)	124.9 (54.7)	143.4 (63.5)	120.8 (50.4)	128.7 (59.9)
BMI (kg/m^2^)	22.7 (4.9)	25.0 (5.2)	25.4 (6.4)	26.0 (6.1)	26.3 (5.2)	26.4 (5.2)	24.4 (6.0)	25.3 (5.9)
WC (cm)	80 (13)	87 (15)	89 (16)	90 (18)	93 (18)	94 (17)	83 (15)	88 (17)
WHR	0.83 (0.11)	0.86 (0.13)	0.88 (0.13)	0.90 (0.13)	0.94 (0.13)	0.94 (0.11)	0.82 (0.11)	0.88 (0.14)
hs-CRP (μg/L)	1.0 (0.97)	1.0 (1.3)	1.1 (1.4)	1.3 (1.5)	1.4 (1.6)	1.1 (1.1)	1.2 (1.6)	1.2 (1.4)
SII	334.6 (202.1)	390.0 (211.6)	383.8 (224.7)	364.9 (225.4)	360.2 (223.6)	360.9 (204.6)	374.6 (229.8)	370.2 (218.0)
Insulin (μIU/mL*)	6.6 (3.2)	6.5 (4.1)	6.8 (4.5)	7.4 (5.9)	7.7 (5.4)	7.7 (5.7)	6.3 (4.2)	7.0 (4.8)
HOMA-IR	1.35 (0.81)	1.38 (0.95)	1.46 (1.13)	1.68 (1.49)	1.82 (1.6)	1.73 (1.47)	1.35 (1.)	1.51 (1.22)
HbA1c (%)	3.3 (0.5)	3.4 (0.5)	3.6 (0.5)	3.8 (0.6)	3.9 (0.5)	3.6 (0.5)	3.6 (0.6)	3.6 (0.6)
FBG (mg/dL)	85 (11.5)	87 (10)	89 (13)	91 (14)	93 (16.5)	92 (13)	87 (12)	89 (13)
Apo A (mg/L)	161 (40.5)	159 (35)	164 (37)	169 (41)	173.5 (37.5)	153 (30)	179 (38)	164.5 (39)
Apo B (mg/L)	82 (24.5)	91 (32)	96 (31)	98 (25)	96 (25.75)	98 (29)	90 (27)	94 (29)
TG (mg/dL)	75 (42.5)	86 (62.75)	85 (59)	94 (58.5)	93 (51)	102 (68)	79 (44.75)	88 (56)
Total cholesterol (mg/dL)	186 (49)	196 (45.5)	207 (52)	210 (47)	205 (52.5)	201 (52)	203 (48)	202 (50)
LDL-c (mg/dL)	106 (38)	121 (46)	128 (48)	128 (44)	122 (46)	126 (45)	122 (46)	124 (45)
HDL-c (mg/dL)	57 (20)	54 (16)	57 (18)	57 (21)	58 (21.5)	50 (15)	63 (17)	57 (19)
Urinary microalbumin (mg/L)	6.5 (6.5)	7.6 (7.1)	6.5 (6.6)	6.1 (6.4)	8.4 (9.8)	7.2 (7.1)	6.5 (6.7)	6.9 (6.9)
Urinary creatinine (μM)	165 (109)	171 (104)	146 (89)	119 (92)	116 (66)	166 (100)	118 (87)	141 (100)
Albumin/creatinine	5.4 (4.7)	5.1 (4.6)	6.0 (4.9)	6.3 (6.2)	8.8 (9.1)	4.8 (5.3)	7.1 (6.2)	6.1 (5.4)
Uric acid in serum (mg/dL)	4.9 (1.9)	5.0 (1.8)	5.0 (1.7)	5.3 (1.6)	5.4 (1.7)	5.9 (1.4)	4.5 (1.2)	5.1 (1.7)
25-OH Vitamin D (ng/mL)	21.8 (13.0)	22.0 (14.4)	23.9 (14.7)	26.7 (13.5)	27.7 (13.8)	22.0 (13.5)	26.8 (14.0)	24.6 (14.4)
Calcium in serum (mg/dL)	9.3 (0.4)	9.2 (0.4)	9.3 (0.4)	9.3 (0.4)	9.3 (0.4)	9.3 (0.4)	9.2 (0.4)	9.3 (0.4)
Urinary sodium (mg/dL)	95 (67.75)	104 (62)	96 (62)	90.5 (62)	98 (56.5)	106 (64.5)	90 (59)	97 (63)
Sodium in serum (mg/dL)	140 (2)	140 (2)	141 (2)	141 (2)	141 (2)	141 (2)	141 (3)	141 (2)
Potassium serum (mg/dL)	4.0 (0.3)	4.1 (0.4)	4.1 (0.3)	4.1 (0.4)	4. 0 (0.4)	4.1 (0.3)	4.1 (0.3)	4.1 (0.3)
Magnesium in serum (mg/dL)	2.0 (0.2)	2.0 (0.1)	2.0 (0.1)	2.0 (0.1)	2.0 (0.1)	2.0 (0.1)	2.0 (0.1)	2.0 (0.1)
Ferritin (ng/mL)	78.0 (118.2)	82.3 (151.9)	99.5 (125.7)	117.2 (131.3)	139.7 (157.8)	165.6 (172.2)	67.2 (82.8)	104.2 (139.1)
Hematocrit (%)	42.6 (6.1)	43.1 (5.2)	42.9 (5.2)	42.7 (4.9)	43.3 (4.8)	45.6 (3.6)	40.8 (3.3)	43.0 (5.2)
Hemoglobin (g/L)	14.2 (2.2)	14.3 (2.1)	14.1 (1.9)	14.2 (1.8)	14.3 (1.8)	15.2 (1.3)	13.4 (1.1)	14.2 (1.9)
TSH (mIU/L)	1.7 (1.0)	1.8 (1.1)	1.8 (1.0)	1.6 (1.0)	1.7 (1.0)	1.7 (1.0)	1.7 (1.2)	1.7 (1.0)
Free T3 (pmol/L)	2.7 (0.5)	2.6 (0.4)	2.6 (0.4)	2.6 (0.4)	2.6 (0.4)	2.7 (0.4)	2.6 (0.4)	2.6 (0.4)
Free T4 (ng/dL)	0.98 (0.57)	0.97 (0.14)	0.97 (0.14)	0.97 (0.15)	1.0 (0.16)	0.97 (0.14)	0.97 (0.14)	0.97 (14)
SBP (mmHg)	115.5 (16.5)	117.5 (20.0)	124 (20.1)	127 (20.4)	136.2 (23.6)	129.0 (19.2)	117.5 (22.0)	123.0 (22.0)
CSBP (mmHg)	108.5 (17.7)	112.0 (20.0)	116.8 (20.0)	122.0 (20.0)	127.0 (23.0)	122.0 (20.0)	112.0 (21.2)	117.0 (21.7)
DBP (mmHg)	72.7 (12.5)	76.0 (13.5)	80.0 (14.5)	80.5 (13.0)	80.2 (15.0)	81.0 (13.1)	76.0 (14.0)	78.0 (14.0)
CDBP (mmHg)	71.0 (9.7)	75.0 (13.0)	79.0 (13.0)	80.0 (11.5)	80.0 (11.5)	80.0 (11.0)	75.0 (13.0)	78.0 (13.0)
GFR^a^ (ml/min/1.73m^2^)	93.2 (16.9)	85.5 (14.1)	83.5 (15.9)	80.3 (14.7)	75.5 (16.9)	86.5 (19.8)	81.5 (13.9)	83.2 (16.9)
PWV (m/s)	6.7 (1.4)	7.1 (2.0)	7.5 (1.7)	8.2 (2.3)	9.5 (3.4)	7.8 (2.3)	7.5 (2.3)	7.6 (2.3)
Vascular age (years)	37 (18)	40 (22)	43 (18)	52 (19)	63 (23)	48 (22)	45 (23)	47 (22)

*AHEI* Alternative Healthy Eating Index, *MDS* Mediterranean Diet Score, *DASH-S* Dietary Approaches to Stop Hypertension Score, *DQI-I* Diet Quality Index-International, *DII* Dietary Inflammatory Index, *DAI* Dietary Antioxidant Index, *NNRS* Naturally Nutrient-Rich Score, *SII* Systemic Immune-Inflammation Index, *BMI* Body Mass Index, *WC* Waist Circumference, *hs-CRP* high-sensitivity C-reactive protein, *HOMA-IR* Homeostatic Model Assessment for Insulin Resistance, *FBG* Fasting blood glucose, *TG* Triglycerides, *LDL-c* Low-density lipoprotein cholesterol, *HDL-c* High-density lipoprotein cholesterol, *TSH* Thyroid-stimulating hormone, *GFR* Glomerular filtration rate, *CSBP* Central systolic blood pressure, *CDBP* Central diastolic blood pressure, *PWV* Carotid-femoral pulse wave velocity

^a^Estimated by Modification of Diet in Renal Disease (MDRD) method

*μIU/mL = 6.00 pmol/L

### Correlations

**Fig. 1:**
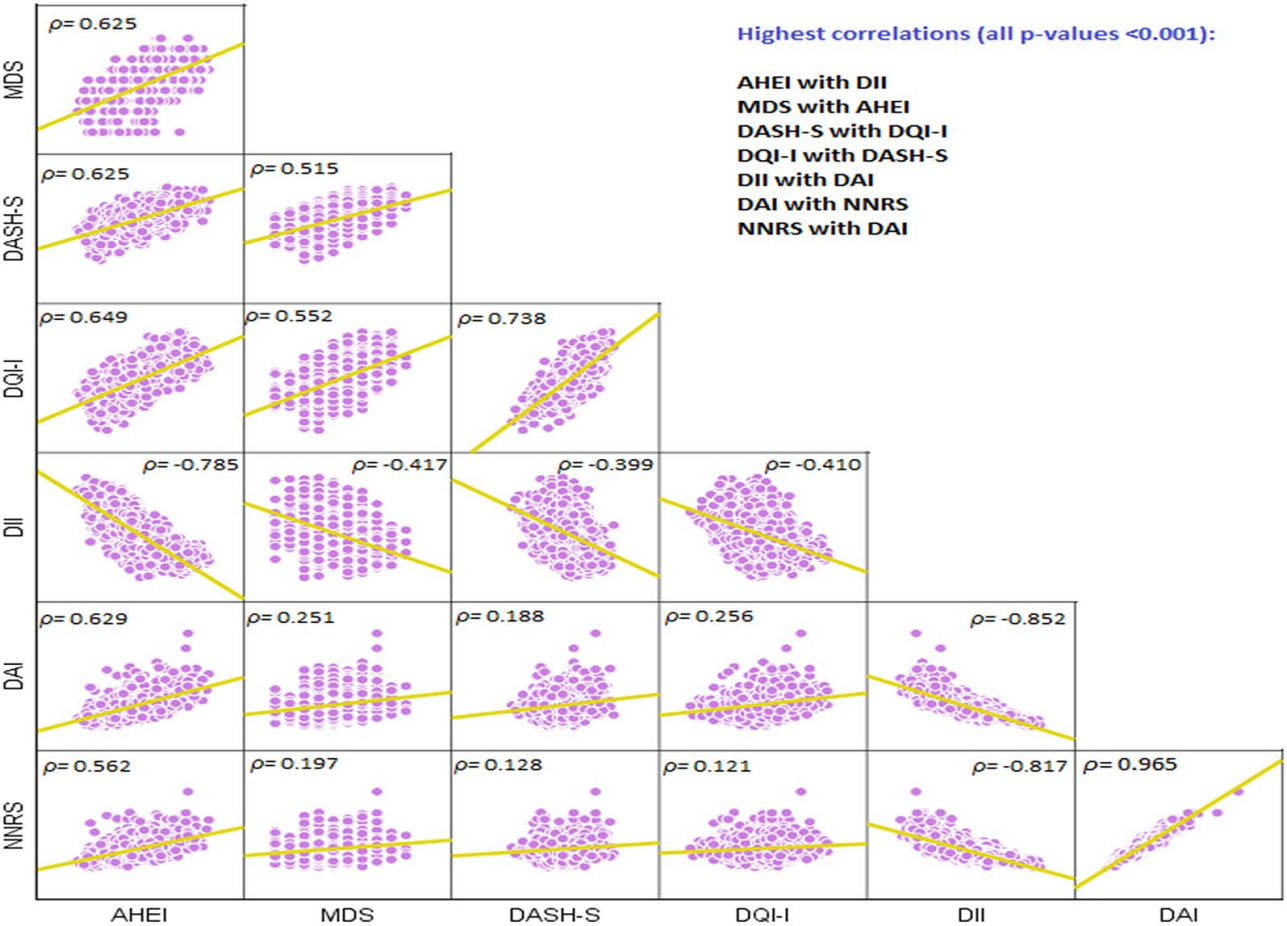
Spearman correlation (*ρ*) between the investigated dietary quality indices and respective linear trendlines. *AHEI* Alternative Healthy Eating Index, *MDS* Mediterranean Diet Score, *DASH-S* Dietary Approaches to Stop Hypertension Score, *DQI-I* Diet Quality Index-International, *DII* Dietary Inflammatory Index, *DAI* Dietary Antioxidant Index, *NNRS* Naturally Nutrient-Rich Score

Spearman correlation (ρ (rho)) between the investigated dietary quality indices and respective linear trendlines are shown in Fig. 1. According to Spearman correlation analyses, all dietary indices significantly correlated with one another (Fig. 1).

In addition, the Spearman correlation between the investigated dietary indices and daily nutrient intakes is presented in Table 4. The largest number of significant correlations (*ρ* > 0.50) between dietary indices and nutrient intakes (36 nutrients in total) was found for the NNRS (34 nutrients), followed by the DAI (33 nutrients), DII (31 nutrients), AHEI (13 nutrients), DQI-I (4 nutrients), DASH-S (2 nutrients), and MDS (1 nutrient). Strongest correlations between dietary indices and nutrients were AHEI with total dietary fiber (*ρ* = 0.827); MDS with soluble dietary fiber (*ρ* = 0.524); DASH-S with total dietary fiber (*ρ* = 0.540); DQI-I with vitamin C (*ρ* = 0.633); DII with folate (*ρ* = − 0.853); DAI (*ρ* = 0.900) and NNRS (*ρ* = 0.923) with phosphorus (Table 4). Also, the Spearman correlation between the investigated dietary indices and daily food group intakes (14 groups in total) is shown in Table 5. The indices correlating (*ρ* > 0.50) significantly with most food groups were DII (3 groups), DAI (3 groups), and NNRS (3 groups), followed by AHEI (2 groups) and DASH-S (2 groups) (Table 5). The highest Spearman correlations between dietary indices and food groups included AHEI (*ρ* = 0.705), MDS (*ρ* = 0.554), and DII (*ρ* = -0.649) with vegetables; DASH-S (*ρ* = 0.515) and DQI-I (*ρ* = 0.613) with fruits; DAI (*ρ* = 0.683) and NNRS (*ρ* = 0.719) with protein-rich foods (Table 5). In addition, significant correlations (*p* value < 0.001) were found between protein-rich foods, fast foods, red meat group, fish group, lipids, sugary products with NNRS; grains, and starchy vegetables with DAI; fruits and vegetables with AHEI; dairy group, and sugar-sweetened beverages with DASH-S; non-caloric beverages with DII: and alcoholic beverages with DQI-I (Table 5).

**Table 4:** Spearman correlation (*ρ)* between the investigated dietary indices and daily nutrient intakes per capita from the ORISCAV-LUX 2 study

Nutrients	AHEI		MDS		DASH-S		DQI-I		DII		DAI		NNRS	
	*ρ*	*p* value	*ρ*	*p* value	*ρ*	*p* value	*ρ*	*p* value	*ρ*	*p* value	*ρ*	*p* value	*ρ*	*p* value
Total fat (g/d)	0.345	< 0.001	0.035	0.186	− 0.013	0.632	− 0.207	< 0.001	**− 0.570**	** < 0.001**	**0.738**	** < 0.001**	0.798*	** < 0.001**
Cholesterol (mg/d)	0.150	< 0.001	− 0.109	< 0.001	− 0.229	< 0.001	− 0.306	< 0.001	− 0.468	< 0.001	**0.689**	** < 0.001**	0.753*	** < 0.001**
SFA (g/d)	0.166	< 0.001	− 0.142	< 0.001	− 0.093	< 0.001	− 0.274	< 0.001	− 0.441	< 0.001	**0.665**	** < 0.001**	0.717*	** < 0.001**
MUFA (g/d)	0.334	< 0.001	0.088	0.001	− 0.045	0.090	− 0.217	< 0.001	**− 0.538**	** < 0.001**	**0.698**	** < 0.001**	0.775*	** < 0.001**
PUFA (g/d)	0.470	< 0.001	0.145	< 0.001	0.101	< 0.001	− 0.082	0.002	**− 0.599**	** < 0.001**	**0.638**	** < 0.001**	0.661*	** < 0.001**
Total protein (g/d)	0.346	< 0.001	0.018	0.507	− 0.079	0.003	− 0.050	0.061	**− 0.660**	** < 0.001**	**0.861**	** < 0.001**	0.891*	** < 0.001**
Animal-based protein (g/d)	0.134	< 0.001	− 0.115	< 0.001	− 0.242	< 0.001	− 0.239	< 0.001	− 0.496	< 0.001	**0.711**	** < 0.001**	0.766*	** < 0.001**
Vegetable protein (g/d)	**0.679**	** < 0.001**	0.341	< 0.001	0.353	< 0.001	0.418	< 0.001	**− 0.714**	** < 0.001**	**0.792**^**c**^	** < 0.001**	**0.687**	** < 0.001**
Total carbohydrates (g/d)	0.476	< 0.001	0.138	< 0.001	0.185	< 0.001	0.296	< 0.001	**− 0.577**	** < 0.001**	**0.707**^**c**^	** < 0.001**	**0.694**	** < 0.001**
Total dietary fiber (g/d)	***0.827***^**a, &**^	** < 0.001**	0.497	< 0.001	***0.540***^***&***^	** < 0.001**	**0.628**	** < 0.001**	**− 0.802**	** < 0.001**	**0.741**	** < 0.001**	**0.670**	** < 0.001**
Soluble dietary fiber (g/d)	**0.813**^**a**^	** < 0.001**	***0.524***^***&***^	** < 0.001**	**0.522**	** < 0.001**	**0.615**	** < 0.001**	**− 0.767**	** < 0.001**	**0.700**	** < 0.001**	**0.627**	** < 0.001**
Total phenolics (mg/d)	**0.581**^**a**^	** < 0.001**	0.341	< 0.001	0.453	< 0.001	0.403	< 0.001	**− 0.566**	** < 0.001**	0.499	< 0.001	0.438	< 0.001
Added sugars (g/d)	0.035	0.189	− 0.153	< 0.001	− 0.053	0.047	− 0.062	0.019	− 0.145	< 0.001	0.281	< 0.001	0.306*	< 0.001
Simple sugars (g/d)	0.498	< 0.001	0.153	< 0.001	0.316	< 0.001	0.386	< 0.001	**− 0.547**	** < 0.001**	**0.625**^**c**^	** < 0.001**	**0.604**	** < 0.001**
Beta-carotene (µg/d)	**0.601**	** < 0.001**	0.404	< 0.001	0.427	< 0.001	0.437	< 0.001	**− 0.652**^**b**^	** < 0.001**	0.467	< 0.001	0.416	< 0.001
Vitamin A (µg/d)	0.135	< 0.001	− 0.107	< 0.001	− 0.114	< 0.001	− 0.291	< 0.001	− 0.416	< 0.001	**0.606**	** < 0.001**	0.669*	** < 0.001**
Vitamin D (µg/d)	0.300	< 0.001	0.151	< 0.001	0.008	0.767	− 0.088	0.001	**− 0.532**	** < 0.001**	**0.515**	** < 0.001**	0.583*	** < 0.001**
Vitamin E (mg/d)	0.427	< 0.001	0.156	< 0.001	0.054	0.044	− 0.097	< 0.001	**− 0.532**	** < 0.001**	**0.621**	** < 0.001**	0.656*	** < 0.001**
Vitamin K (µg/d)	**0.638**	** < 0.001**	0.439	< 0.001	0.374	< 0.001	0.362	< 0.001	**− 0.652**^**b**^	< 0.001	**0.539**	** < 0.001**	**0.509**	** < 0.001**
Vitamin C (mg/d)	**0.705**^**a**^	** < 0.001**	0.467	< 0.001	0.498	< 0.001	***0.633***^***&***^	** < 0.001**	**− 0.687**	** < 0.001**	**0.649**	** < 0.001**	**0.551**	** < 0.001**
Thiamine (B1) (mg/d)	0.473	< 0.001	0.124	< 0.001	0.106	< 0.001	0.193	< 0.001	**− 0.718**	** < 0.001**	**0.852**	** < 0.001**	0.864*	** < 0.001**
Riboflavin (B2) (mg/d)	0.428	< 0.001	0.025	0.345	0.162	< 0.001	0.161	< 0.001	**− 0.696**	** < 0.001**	**0.818**	** < 0.001**	0.866*	** < 0.001**
Niacin (B3) (mg/d)	0.358	< 0.001	0.039	0.141	− 0.041	0.122	0.066	0.013	**− 0.661**	** < 0.001**	**0.802**	** < 0.001**	0.832*	** < 0.001**
Pantothenic acid (B5) (mg/d)	**0.550**	** < 0.001**	0.152	< 0.001	0.224	< 0.001	0.255	< 0.001	**− 0.775**	** < 0.001**	**0.870**	** < 0.001**	0.896*	** < 0.001**
Vitamin (B6) (mg/d)	**0.569**	** < 0.001**	0.201	< 0.001	0.210	< 0.001	0.292	< 0.001	**− 0.785**	** < 0.001**	**0.833**	** < 0.001**	0.850*	** < 0.001**
Folate (B9) (µg/d)	**0.813**	** < 0.001**	0.436	< 0.001	0.499	< 0.001	**0.552**	** < 0.001**	**− *0.853***^**b,&**^	** < 0.001**	**0.805**	** < 0.001**	**0.770**	** < 0.001**
Vitamin (B12) (µg/d)	0.236	< 0.001	0.057	0.033	− 0.141	< 0.001	− 0.163	< 0.001	**− 0.532**	** < 0.001**	**0.668**	** < 0.001**	0.770*	** < 0.001**
Iron (mg/d)	**0.579**	** < 0.001**	0.205	< 0.001	0.166	< 0.001	0.233	< 0.001	**− 0.800**	** < 0.001**	**0.890**	** < 0.001**	0.900*	** < 0.001**
Magnesium (mg/d)	**0.685**	** < 0.001**	0.280	< 0.001	0.325	< 0.001	0.344	< 0.001	**− 0.840**	** < 0.001**	**0.888**^**c**^	** < 0.001**	**0.851**	** < 0.001**
Zinc (mg/d)	0.384	< 0.001	0.040	0.134	− 0.014	0.606	0.035	0.190	**− 0.678**	** < 0.001**	**0.871**^**c**^	** < 0.001**	**0.863**	** < 0.001**
Selenium (µg/d)	0.429	< 0.001	0.168	< 0.001	− 0.014	0.596	0.027	0.316	**− 0.692**	** < 0.001**	**0.828**	** < 0.001**	0.835*	** < 0.001**
Calcium (mg/d)	0.458	< 0.001	0.079	0.003	0.305	< 0.001	0.238	< 0.001	**− 0.634**	** < 0.001**	**0.740**^**c**^	** < 0.001**	**0.726**	** < 0.001**
Iodine (µg/d)	0.382	< 0.001	0.095	< 0.001	0.078	0.003	0.012	0.644	**− 0.601**	** < 0.001**	**0.725**	** < 0.001**	0.776*	** < 0.001**
Potassium (mg/d)	**0.681**	** < 0.001**	0.294	< 0.001	0.329	< 0.001	0.380	< 0.001	**− 0.835**	** < 0.001**	**0.884**^***c***^	** < 0.001**	**0.859**	** < 0.001**
Phosphorus (mg/d)	0.455	< 0.001	0.071	0.008	0.077	0.004	0.062	0.019	**− 0.735**	** < 0.001**	***0.900***^***&***^	** < 0.001**	0.923^*,&^	** < 0.001**
Sodium (mg/d)	0.265	< 0.001	− 0.017	0.534	− 0.165	< 0.001	− 0.109	< 0.001	**− 0.553**	** < 0.001**	**0.737**	** < 0.001**	0.759*	** < 0.001**
N of correlations over 0.50	**13**		**1**		**2**		**4**		**31**		**33**		**34**	

Correlations higher than 0.50 are shown in Bold

Highest correlations (in each column) between dietary indices and nutrients are shown in italics. Highest correlations (in each row) between nutrients and dietary indices are given underlined

*AHEI* Alternative Healthy Eating Index, *MDS* Mediterranean Diet Score, *DASH-S* Dietary Approaches to Stop Hypertension Score, *DQI-I* Diet Quality Index-International, *DII* Dietary Inflammatory Index, *DAI* Dietary Antioxidant Index, *NNRS* Naturally Nutrient-Rich Score, *ρ* Spearman's rho

^&^Strongest correlation (per column) of HEI with total dietary fiber; MDS with soluble dietary fiber; DASH-S with total dietary fiber; DQI-I with vitamin C; DII with folate; DAI and NNRS with phosphorus

*Total fat, cholesterol, SFA, MUFA, PUFA, total protein, animal-based protein, added sugar, vitamin A, D, E, B1, B2, B3, B5, B6, B12, iron, selenium, iodine, phosphorus, and sodium with NNRS

^a^Total dietary fiber, soluble dietary fiber, total phenolics, and vitamin C with AHEI

^b^Beta-carotene, vitamin K, and folate with DII

^c^Vegetable protein, total carbohydrates, simple sugars, magnesium, zinc, calcium, and potassium with DAI

**Table 5:** Spearman correlation (*ρ)* between the investigated dietary indices and daily food group intakes from the ORISCAV-Lux 2 study (correlations higher than 0.50 are shown in bold)

Food groups	AHEI		MDS		DASH-S		DQI-I		DII		DAI		NNRS	
	*ρ*	*p* value	*ρ*	*p* value	*ρ*	*p* value	*ρ*	*p* value	*ρ*	*p* value	*ρ*	*p* value	*ρ*	*p* value
Grains (g/d)	0.310	< 0.001	0.153	< 0.001	0.258	< 0.001	0.309	< 0.001	− 0.346	< 0.001	**0.377**^**a**^	< 0.001	0.348	< 0.001
Fruits (g/d)	**0.629**^**b**^	**< 0.001**	0.422	< 0.001	***0.515***^***&***^	**< 0.001**	***0.613***^***&***^	**< 0.001**	**− 0.500**	**< 0.001**	0.476	< 0.001	0.403	< 0.001
Vegetables (g/d)	***0.705***^**b,&**^	**< 0.001**	***0.554***^***&***^	**< 0.001**	**0.510**	**< 0.001**	0.494	< 0.001	***− 0.649***^***&***^	**< 0.001**	0.483	< 0.001	0.435	< 0.001
Starchy vegetables (g/d)	0.107	< 0.001	− 0.010	0.717	− 0.062	0.020	0.026	0.336	− 0.206	< 0.001	**0.250**^**a**^	< 0.001	0.246	< 0.001
Protein-rich foods (g/d)	0.231	< 0.001	0.028	0.296	− 0.213	< 0.001	− 0.189	< 0.001	**− 0.534**	**< 0.001**	***0.683***^***&***^	**< 0.001**	0.719^*,&^	**< 0.001**
Fast foods (g/d)	0.057	0.033	− 0.092	0.001	− 0.274	< 0.001	− 0.259	< 0.001	− 0.291	< 0.001	0.436	< 0.001	0.474*	**< 0.001**
Red meat group (g/d)	− 0.077	0.004	− 0.245	< 0.001	− 0.451	< 0.001	− 0.343	< 0.001	− 0.280	< 0.001	**0.512**	**< 0.001**	0.549*	**< 0.001**
Fish group (g/d)	0.314	< 0.001	0.291	< 0.001	− 0.010	0.708	− 0.056	0.037	− 0.451	< 0.001	0.449	< 0.001	0.500*	**< 0.001**
Dairy (mL/d)	0.134	< 0.001	− 0.181	< 0.001	**0.345**^**c**^	< 0.001	0.160	< 0.001	− 0.257	< 0.001	0.298	< 0.001	0.326	< 0.001
Lipids (g/d)	0.110	< 0.001	− 0.063	0.018	− 0.122	< 0.001	− 0.326	< 0.001	− 0.253	< 0.001	0.393	< 0.001	0.453*	< 0.001
Sugary products (g/d)	0.089	0.001	− 0.066	0.013	− 0.007	0.792	− 0.007	0.800	− 0.138	< 0.001	0.225	< 0.001	0.228*	< 0.001
Non-caloric beverages (mL/d)	0.204	< 0.001	0.097	< 0.001	0.124	< 0.001	0.136	< 0.001	**− 0.255**^**d**^	< 0.001	0.245	< 0.001	0.179	< 0.001
Sugar-sweetened beverages (mL/d)	0.049	0.068	0.024	0.374	**− 0.306**^**c**^	< 0.001	− 0.046	0.083	− 0.078	0.003	0.109	< 0.001	0.108	< 0.001
Alcoholic beverages (mL/d)	0.045	0.092	− 0.239	< 0.001	− 0.170	< 0.001	**− 0.275**^**f**^	< 0.001	− 0.217	< 0.001	0.193	< 0.001	0.230	< 0.001
N of correlations over 0.50	**2**		**1**		**2**		**1**		**3**		**3**		**3**	

Highest correlations (in each column) between dietary indices and food groups are shown in italics. Highest correlations (in each row) between food groups and dietary indices are underlined

*AHEI* Alternate Healthy Eating Index, *MDS* Mediterranean Diet Score, *DASH-S* Dietary Approaches to Stop Hypertension Score, *DQI-I* Diet Quality Index-International, *DII* Dietary Inflammatory Index, *DAI* Dietary Antioxidant Index, *NNRS* Naturally Nutrient-Rich Score, *ρ* Spearman's rho

^&^Strongest correlation per column for AHEI, MDS, and DII with vegetables; DASH-S and DQI-I with fruits; DAI and NNRS with protein-rich foods

*Protein-rich foods, fast foods, red meat group, fish group, lipids, and sugary products with NNRS

^a^Grains, and starchy vegetables with DAI

^b^Fruits and vegetables with AHEI

^c^Dairy group and sugar-sweetened beverages with DASH-S

^d^Non-caloric beverages with DII

^f^Alcoholic beverages with DQI-I

### Regression models

#### Associations between diet quality indices and serum and metabolic biomarkers

Multivariable general linear regression models (adjusted for age, gender, birth country, marital status, education, job, income, IPAQ scoring, and current smoking) of the associations between diet quality indices as continuous variables revealed high significant associations between dietary indices and metabolic biomarkers: between NNRS (Beta = 0.077, 95% CI 0.011, 0.144) and urinary sodium, and DASH-S (Beta = − 2.001, 95% CI − 3.572, − 0.430) with triglycerides (Table 6). According to Table 6, the largest number of significant associations between a Diet Quality Index and a measured metabolic parameter was found for DASH-S (with 14 parameters), followed by the DQI-I (*n* = 13), AHEI (*n* = 11), MDS (*n* = 8), DAI (*n* = 5), NNRS (*n* = 6) and the lowest number for DII, with two parameters. Similar results were obtained, with slightly higher beta-coefficients, when we used quality indices as a categorical variable (i.e., quartiles, Supplementary Table 8).

**Table 6:** Multivariable linear regression^a^ of the associations between diet quality indices (as continuous outcome), serum, and metabolic biomarkers (significant associations are shown in Bold)

	AHEI		MDS		DASH-S		DQI-I		DII		DAI		NNRS	
	β (95% CI)	*p* value	β (95% CI)	*p* value	β (95% CI)	*p* value	β (95% CI)	*p* value	β (95% CI)	*p* value	β (95% CI)	*p* value	β (95% CI)	*p* value
Anthropometry														
BMI (kg/m^2^)	**− 0.039 (− 0.063, − 0.014)**	**0.002**	− 0.259 (− 0.443, − 0.075)	0.006	**− 0.129 (− 0.197, − 0.061)**	** < 0.001**	**− 0.064 (− 0.101, − 0.026)**	**0.001**	0.011 (− 0.150, 0.173)	0.890	0.047 (− 0.002, 0.096)	0.062	**0.007 (0.000, 0.013)**	**0.050**
WC (cm)	**− 0.082 (− 0.144, − 0.019)**	**0.011**	**− 0.607 (− 1.070, − 0.144)**	**0.010**	**− 0.344 (− 0.515, 0.173)**	** < 0.001**	**− 0.163 (− 0.257, − 0.068)**	**0.001**	− 0.170 (− 0.576, 0.236)	0.411	**0.180 (0.056, 0.303)**	**0.005**	**0.026 (0.009, 0.043)**	**0.003**
WHR	**− 0.000 (− 0.001, − 0.000)**	** < 0.001**	− 0.001 (− 0.004, 0.002)	0.372	**− 0.001 (− 0.002, − 0.000)**	** < 0.001**	− 0.000 (− 0.001, 0.000)	0.178	− 0.000 (− 0.002, 0.000)	0.896	− 0.000 (− 0.000, 0.001)	0.203	0.000 (− 0.000, 0.000)	0.214
Inflammation related measurements														
hs-CRP (μg/L)	0.014 (− 0.007, 0.035)	0.199	0.068 (− 0.089, 0.225)	0.396	− 0.018 (− 0.076, 0.041)	0.553	0.022 (− 0.010, 0.054)	0.181	− 0.091 (− 0.227, 0.045)	0.191	**0.052 (0.010, 0.094)**	**0.015**	0.006 (0.000, 0.011)	0.052
SII	− 0.448 (− 1.781, 0.885)	0.510	− 3.551 (− 13.43, 6.328)	0.481	− 1.842 (− 5.528, 1.845)	0.327	− 0.848 (− 2.859, 1.164)	0.408	3.881 (− 4.725, 12.49)*	0.376	− 0.405 (− 3.048, 2.238)	0.764	− 0.012 (− 0.371, 0.348)	0.949
Glucose-related measurements														
Insulin (μIU/mL**)	− 0.034 (− 0.095, 0.027)	0.278	− 0.202 (− 0.657, 0.252)	0.382	− 0.030 (− 0.199, 0.139)	0.729	− 0.017 (− 0.110, 0.075)	0.714	0.031 (− 0.364, 0.426)	0.876	0.054 (− 0.068, 0.175)	0.384	0.008 (− 0.009, 0.024)	0.354
HOMA-IR	− 0.143 (− 0.462, 0.177)	0.381	− 1.036 (− 3.403, 1.332)	0.391	− 0.080 (− 0.967, 0.808)	0.861	− 0.060 (− 0.544, 0.425)	0.809	− 0.039 (− 2.104, 2.026)	0.970	0.336 (− 0.307, 0.979)	0.306	0.045 (− 0.043, 0.132)	0.316
HbA1c (%)	0.015 (− 0.008, 0.038)	0.194	0.033 (− 0.139, 0.205)	0.705	0.036 (− 0.028, 0.100)	0.268	0.034 (− 0.001, 0.069)	0.057	− 0.123 (− 0.272, 0.026)	0.106	**0.049 (0.003, 0.095)**	**0.036**	0.005 (− 0.001, 0.011)	0.102
FBG (mg/dL)	0.007 (− 0.058, 0.072)	0.836	− 0.332 (− 0.813, 0.150)	0.177	− 0.058 (− 0.237, 0.121)	0.526	− 0.018 (− 0.116, 0.080)	0.721	− 0.344 (− 0.762, 0.075)	0.107	0.166 (0.037, 0.294)	0.011	**0.019 (0.002, 0.037)**	**0.032**
Lipid-related measurements														
Apo A (mg/L)	**− 0.145 (− 0.286, − 0.004)**	**0.044**	**− 1.061 (− 2.110, − 0.012)**	**0.047**	− 0.288 (− 0.680, 0.104)	0.150	**− 0.313 (− 0.526, − 0.100)**	**0.004**	0.513 (− 0.398, 1.424)	0.269	− 0.253 (− 0.533, 0.027)	0.077	− 0.035 (− 0.073, 0.003)	0.073
Apo B (mg/L)	− 0.065 (− 0.187, 0.056)	0.291	− 0.006 (− 0.910, 0.898)	0.990	**− 0.386 (− 0.722, − 0.049)**	**0.025**	**− 0.194 (− 0.378, − 0.011)**	**0.038**	− 0.570 (− 1.353, 0.213)	0.153	0.225 (− 0.015, 0.466)	0.067	0.030 (− 0.003, 0.063)	0.072
TG (mg/dL)	− 0.566 (− 1.136, 0.004)	0.052	− 1.621 (− 5.855, 2.614)	0.453	***− 2.001 (− 3.572, − 0.430)***	**0.013**	− 0.611 (− 1.473, 0.252)	0.165	2.641 (− 1.036, 6.317)	0.159	− 0.266 (− 1.398, 0.867)	0.645	− 0.032 (− 0.186, 0.122)	0.681
Total cholesterol (mmol/L)	− 0.130 (− 0.341, 0.081)	0.228	− 0.081 (− 1.648, 1.487)	0.920	− 0.497 (− 1.080, 0.085)	0.094	**− 0.391 (− 0.709, − 0.073)**	**0.016**	− 0.848 (− 2.209, 0.513)	0.222	0.238 (− 0.181, 0.657)	0.265	0.034 (− 0.023, 0.091)	0.246
LDL-c (mg/dL)	− 0.043 (− 0.230, 0.143)	0.649	0.376 (− 1.006, 1.758)	0.593	− 0.323 (− 0.837, 0.191)	0.218	− 0.233 (− 0.514, 0.049)	0.105	− 1.101 (− 2.301, 0.100)	0.072	0.340 (− 0.029, 0.709)	0.071	0.047 (− 0.003, 0.097)	0.068
HDL-c (mg/dL)	0.009 (− 0.061, 0.079)	0.797	− 0.044 (− 0.560, 0.472)	0.866	0.143 (− 0.049, 0.335)	0.143	− 0.045 (− 0.150, 0.060)	0.403	− 0.063 (− 0.512, 0.386)	0.782	− 0.101 (− 0.239, 0.036)	0.149	− 0.013 (− 0.032, 0.006)	0.173
Kidney-related measurements														
Urinary microalbumin (mg/L)	− 0.202 (− 0.578, 0.174)	0.292	− 0.995 (− 3.792, 1.802)	0.485	0.187 (− 0.852, 1.226)	0.724	− 0.158 (− 0.725, 0.409)	0.585	2.278 (− 0.128, 4.684)	0.063	− 0.391 (− 1.136, 0.354)	0.303	− 0.048 (− 0.149, 0.053)	0.355
Urinary creatinine (μM)	***− 0.952 (− 1.350, − 0.555)***	** < 0.001**	***− 5.397 (− 8.370, − 2.425)***	** < 0.001**	**− 1.672 (− 2.779, − 0.566)**	**0.003**	***− 1.074 (− 1.677, − 0.472)***	** < 0.001**	***4.348 (1.784, 6.912)***	**0.001**	***− 0.869 (− 1.665, − 0.073)***	**0.032**	− 0.081 (− 0.190, 0.027)	0.142
Albumin/creatinine	− 0.335 (− 0.876, 0.206)	0.225	− 1.858 (− 5.767, 2.050)	0.351	− 0.271 (− 1.738, 1.198)	0.717	− 0.621 (− 1.434, 0.192)	0.134	3.308 (− 0.164, 6.781)	0.062	− 0.572 (− 1.681, 0.538)	0.312	− 0.058 (− 0.209, 0.093)	0.449
Serum uric acid (mg/dL)	**− 0.008 (− 0.014, − 0.002)**	**0.008**	− 0.041 (− 0.086, 0.005)	0.079	**− 0.036 (− 0.052, − 0.019)**	** < 0.001**	**− 0.013 (− 0.022, − 0.004)**	**0.006**	− 0.008 (− 0.047, 0.031)	0.692	0.006 (− 0.006, 0.018)	0.331	0.001 (− 0.001, 0.002)	0.331
Nutritional status														
25-OH Vitamin D in serum (ng/mL)	**0.150 (0.090, 0.210)**	** < 0.001**	0.432 (− 0.019, 0.884)	0.061	**0.266 (0.098, 0.434)**	**0.002**	**0.158 (0.066, 0.249)**	**0.001**	**− 0.629 (− 1.019, − 0.239)**	**0.002**	0.120 (− 0.000, 0.240)	**0.051**	0.010 (− 0.006, 0.027)	0.218
Calcium in serum (mg/dL)	0.001 (− 0.001, 0.003)	0.274	0.011 (− 0.005, 0.027)	0.176	0.002 (− 0.004, 0.008)	0.584	0.002 (− 0.001, 0.005)	0.230	− 0.000 (− 0.014, 0.013)	0.972	− 0.001 (− 0.005, 0.004)	0.779	− 0.000 (− 0.001, 0.000)	0.235
Urinary sodium (mg/dL)	− 0.167 (− 0.413, 0.079)	0.184	− 0.923 (− 2.754, 0.908)	0.323	**− 1.179 (− 1.855, − 0.504)**	**0.001**	**− 0.596 (− 0.966, − 0.227)**	**0.002**	0.725 (− 0.855, 2.305)	0.369	0.335 (− 0.154, 0.825)	0.179	***0.077 (0.011, 0.144)***	**0.023**
Sodium in serum (mg/dL)	0.009 (− 0.001, 0.019)	0.076	0.052 (− 0.023, 0.127)	0.176	− 0.005 (− 0.033, 0.023)	0.734	0.005 (− 0.010, 0.020)	0.526	− 0.048 (− 0.113, 0.017)	0.147	0.020 (0.000, 0.040)	0.049	**0.003 (0.000, 0.006)**	**0.032**
Potassium serum (mg/dL)	− 0.000 (− 0.002, 0.001)	0.740	− 0.002 (− 0.014, 0.011)	0.779	− 0.002 (− 0.007, 0.002)	0.318	− 0.002 (− 0.004, 0.001)	0.209	− 0.002 (− 0.012, 0.009)	0.777	0.002 (− 0.001, 0.005)	0.243	0.000 (− 0.000, 0.001)	0.122
Magnesium in serum (mg/dL)	0.001 (− 0.000, 0.001)	0.112	0.004 (− 0.002, 0.010)	0.206	0.001 (− 0.001, 0.004)	0.267	0.001 (− 0.000, 0.002)	0.091	− 0.000 (− 0.005, 0.005)	0.963	− 0.001 (− 0.002, 0.001)	0.463	− 0.000 (− 0.000, 0.000)	0.234
Ferritin (ng/mL)	− 0.255 (− 0.941, 0.432)	0.467	2.290 (− 2.812, 7.393)	0.379	**− 1.967 (− 3.867, − 0.066)**	**0.043**	− 0.661 (− 1.699, 0.377)	0.212	− 1.173 (− 5.600, 3.254)	0.603	0.297 (− 1.066, 1.659)	0.669	0.037 (− 0.148, 0.222)	0.694
Hematocrit (%)	− 0.003 (− 0.018, 0.012)	0.963	− 0.028 (− 0.137, 0.080)	0.607	− 0.020 (− 0.060, 0.021)	0.341	− 0.007 (− 0.029, 0.015)	0.517	− 0.008 (− 0.103, 0.086)	0.862	0.007 (− 0.022, 0.036)	0.620	0.002 (− 0.002, 0.006)	0.402
Hemoglobin (g/L)	− 0.002 (− 0.007, 0.003)	0.423	− 0.023 (− 0.060, 0.014)	0.221	− 0.007 (− 0.020, 0.007)	0.344	− 0.004 (− 0.012, 0.004)	0.259	− 0.000 (− 0.033, 0.032)	0.977	0.002 (− 0.008, 0.012)	0.724	0.000 (− 0.001, 0.002)	0.519
Hormonal status														
TSH (mIU/L)	**− 0.009 (− 0.015, − 0.003)**	**0.003**	**− 0.052 (− 0.096, − 0.008)**	**0.021**	− 0.012 (− 0.029, 0.004)	0.142	− 0.009 (− 0.018, 0.000)	0.052	0.035 (− 0.004, 0.073)	0.077	− 0.006 (− 0.018, 0.006)	0.311	− 0.000 (− 0.002, 0.001)	0.562
Free T3 (pmol/L)	− 0.000 (− 0.003, 0.002)	0.835	− 0.001 (− 0.020, 0.017)	0.891	0.002 (− 0.005, 0.009)	0.628	0.001 (− 0.003, 0.005)	0.615	0.003 (− 0.014, 0.019)	0.750	− 0.000 (− 0.005, 0.005)	0.944	− 0.000 (− 0.001, 0.001)	0.621
Free T4 (ng/dL)	0.001 (− 0.001, 0.003)	0.375	0.002 (− 0.015, 0.019)	0.797	− 0.001 (− 0.008, 0.005)	0.703	− 0.001 (− 0.004, 0.003)	0.774	− 0.009 (− 0.024, 0.006)	0.235	**0.006 (0.001, 0.010)**	**0.015**	**0.001 (0.000, 0.002)**	**0.002**
Vascular function and cardiovascular risk														
SBP (mmHg)	− 0.075 (− 0.156, 0.005)	0.066	**− 1.092 (− 1.683, − 0.500)**	** < 0.001**	**− 0.315 (− 0.535, − 0.095)**	**0.005**	**− 0.152 (− 0.274, − 0.030)**	**0.014**	0.105 (− 0.416, 0.625)	0.693	− 0.040 (− 0.200, 0.119)	0.619	− 0.011 (− 0.032, 0.011)	0.339
CSBP (mmHg)	− 0.078 (− 0.160, 0.004)	0.063	**− 1.173 (− 1.776, − 0.570)**	** < 0.001**	**− 0.300 (− 0.527, − 0.074)**	**0.009**	**− 0.214 (− 0.339, − 0.090)**	**0.001**	0.177 (− 0.353, 0.707)	0.512	− 0.047 (− 0.211, 0.116)	0.570	− 0.006 (− 0.028, 0.016)	0.595
DBP (mmHg)	**− 0.081 (− 0.137, − 0.025)**	**0.005**	**− 0.701 (− 1.114, − 0.288)**	**0.001**	**− 0.206 (− 0.359, − 0.052)**	**0.009**	**− 0.094 (− 0.179, − 0.009)**	**0.031**	0.192 (− 0.172, 0.555)	0.301	− 0.081 (− 0.193, 0.030)	0.151	− 0.015 (− 0.030, 0.000)	0.053
CDBP (mmHg)	**− 0.091 (− 0.141, − 0.041)**	** < 0.001**	**− 0.743 (− 1.112, − 0.374)**	** < 0.001**	**− 0.200 (− 0.338, − 0.061)**	**0.005**	**− 0.131 (− 0.207, − 0.055)**	**0.001**	0.253 (− 0.071, 0.577)	0.126	− 0.078 (− 0.178, 0.022)	0.126	− 0.013 (− 0.027, 0.000)	0.054
GFR^b^ (ml/min/1.73m^2^)	0.017 (− 0.048, 0.083)	0.606	0.193 (− 0.294, 0.679)	0.437	0.096 (− 0.085, 0.278)	0.298	0.033 (− 0.066, 0.132)	0.515	− 0.080 (− 0.502, 0.341)	0.709	− 0.041 (− 0.171, 0.089)	0.536	− 0.006 (− 0.023, 0.012)	0.521
PWV (m/s)	− 0.007 (− 0.036, 0.023)	0.658	0.028 (− 0.187, 0.242)	0.800	− 0.011 (− 0.091, 0.069)	0.788	0.012 (− 0.032, 0.056)	0.591	0.091 (− 0.097, 0.278)	0.342	− 0.014 (− 0.072, 0.043)	0.625	− 0.004 (− 0.012, 0.004)	0.322
Vascular age (years)	**− 0.110 (− 0.203, − 0.018)**	**0.020**	− 0.618 (− 1.303, 0.066)	0.077	− 0.240 (− 0.498, 0.017)	0.067	− 0.072 (− 0.214, 0.070)	0.321	0.458 (− 0.138, 1.053)	0.132	− 0.098 (− 0.281, 0.085)	0.294	− 0.018 (− 0.043, 0.007)	0.162
No. of sign. associations	**11**		**8**		**14**		**13**		**2**		**5**		**6**	

Highest correlations (in each column) between dietary indices and food groups are shown in italics. Highest correlations (in each row) between food groups and dietary indices are underlined

*AHEI* Alternative Healthy Eating Index, *MDS* Mediterranean Diet Score, *DASH-S* Dietary Approaches to Stop Hypertension Score, *DQI-I* Diet Quality Index-International, *DII* Dietary Inflammatory Index, *DAI* Dietary Antioxidant Index, *NNRS* Naturally Nutrient-Rich Score, *SII* Systemic Immune-Inflammation Index, *BMI* Body Mass Index, *WC* Waist Circumference, *hs-CRP* high-sensitivity C-reactive protein, *HOMA-IR* Homeostatic Model Assessment for Insulin Resistance, *FBG* Fasting blood glucose, *TG* Triglycerides, *LDL*-c Low-density lipoprotein cholesterol, *HDL*-c High-density lipoprotein cholesterol, *TSH* Thyroid-stimulating hormone, *GFR* Glomerular Filtration Rate, *CSBP* Central systolic blood pressure, *CDBP* Central diastolic blood pressure, *PWV* Carotid-femoral pulse wave velocity

*E-DII (Beta = 14.392, 95% CI: 4.814–23.970; *p* value = 0.003)

**μIU/mL = 6.00 pmol/L

^a^Adjusted model: age (5 group), gender, birth country, marital status, education, job, income (8 group), IPAQ scoring, current smoking

^b^Estimated by Modification of Diet in Renal Disease (MDRD) method

When looking for combinations of 2 dietary indices that explain the largest number of measured serum and metabolic parameters, the combinations of DASH-S with AHEI (together significantly associated with a total of 18 serum/metabolic parameters), as well as DASH-S with DAI (17) and DASH-S with NNRS (17) were most promising, with both the DAI and the NNRS being nutrient-based indices, compared to the DASH-S (Supplementary Table 9).

In addition, unadjusted multivariable linear regression models of the associations between diet quality indices as continuous and categorical variables and metabolic biomarkers are shown in Supplementary Tables 6 and 7, respectively. As expected, when using dietary indices as quartiles, the results were similar to analyses based on indices as continuous variables, except that the beta-coefficients increased (Supplementary Tables 6 and 7).

## Discussion

In this study, we investigated the association of frequently employed diet quality indices, covering complementary dietary aspects, including nutrient- and food-group-based ones, appertaining to their association with a number of parameters, including biomarkers related to disease risk and/or nutrient status in a rather general adult population. As there are major differences between diet quality indices that are food-group-based (which do not require linking them with food composition databases, introducing another source of variability) and indices that are based on nutrients, we tried to choose indices of both types. Some indices included both aspects (and even three aspects when considering non-nutrients). Our final regression models highlighted that these diet quality indices were associated with different serum and metabolic parameters, such as anthropometry, inflammation, blood glucose, blood lipids, kidney-related parameters, nutritional and hormonal status, and vascular function, with the highest number of significant associations found for the DASH-S, followed by DQI-I, AHEI, MDS, DAI, NNRS and finally the DII.

Nutrition-related diseases are predominantly multifactorial, influenced by the entire array of macro-, micro- and non-nutrients ingested and their interactions [46]. For this purpose, various indices, such as the DASH-S, AHEI, DQI-I, and DAI have been developed and used in research and public health, considering several aspects of the diet. Some of these indices have been validated, such as by measuring their association with serum biomarkers, and their construction criteria and reliability have been examined, with their clinical diagnostic power having been tested for certain populations and certain disease endpoints [9, 47].

In our study, the DASH-S was associated with the largest number of selected parameters (Table 6). A systematic review and meta-analysis of randomized controlled trials reported that adherence to DASH could reduce SBP and DBP [33]. Phillips et al. [48] also examined the association between DASH-S and a large number of cardiometabolic relevant biomarkers, concluding that DASH-S was associated with improved adiposity measures such as BMI and WC and a less insulin-resistant, less pro-thrombotic, less pro-inflammatory, and less pro-atherogenic cardiometabolic profile. In the present study, the significant association of the DASH-S with blood pressure-related biomarkers and sodium excretion shows the validity of this index, as the DASH diet was designed for this purpose. Indeed, dietary sodium intake remains almost 2 times above WHO recommendations of 5 g/d for most Westernized countries [49], being a major cause of elevated blood pressure and cardiovascular-related deaths. According to a recent report [50], over-consumption of dietary sodium is related to 3 million annual deaths globally and 60 million DALYs. In the present study, median sodium excretion was 97 mg/dL, which is likely to represent a higher-than-needed salt intake.

In addition, in line with our results, a recent study that examined the relationship between dietary quality, assessed by DASH-S, and cardiometabolic health biomarkers, concluded that a higher DASH-S was significantly associated with lower BMI, WC, TNF-α, IL-6, white blood count (WBC) and plasminogen activator inhibitor-1 (PAI-1) concentrations, and reduced insulin resistance [48]. In addition, fewer small LDL-c, HDL-c, and VLDL-c particles were observed among those with better DASH-S [48]. Participants in the top DASH-S quartile had a 48% and 54% lower likelihood of metabolic syndrome and central obesity, respectively, than those in the lowest DASH-S quartile [48]. The authors suggested that a high-quality diet assessed with DASH-S was associated with less insulin resistance, improved adiposity measures and favourable pro-inflammatory and pro-atherogenic cardiometabolic profile, and less pro-thrombotic properties and might affect metabolic syndrome and central obesity risk [48]. These findings could have public health and clinical significance regarding dietary approaches to promote cardiometabolic health and warrant further investigations.

Similar to the DASH-S, the highly correlated DQI-I was associated with also most anthropometric markers, urinary sodium, and blood pressure as well as certain blood lipids. The DQI-I score of 64% suggested a rather limited dietary diversity. Similar as for the DQI, Vandevijvere et al. also investigated various aspects of the diet, such as within-food group and overall diversity, and some dimensions of diet quality similar to the DQI-I, such as moderation, adequacy, and balance, derived from the food-based dietary guidelines (FBDG) in Belgium [51], concluding that overall diet diversity derived from the FBDG is a practical benchmark of dietary quality. Another advantage of the DQI-I may be its compromise of being both a food group and nutrient-based index, and such a combination may constitute a more sophisticated manner to assess the overall quality of the diet [52, 53].

Similar findings as for the DQI-I were encountered for the AHEI, which also correlated highly with the DQI-I, though its association with blood pressure markers and sodium intake was less pronounced. Other studies, such as the one by Kim et al., also showed a significant correlation between (among others) DQI-I and AHEI and glycemic status (including HbA1c, FBG, and postprandial 2-h glucose) in Korean patients with T2D [54], which was not found in the present study. It is possible that the different populations or the dietary assessment method influenced the results. Their study estimated dietary intake based on a single 24-h recall method. While such a method may reflect a more current diet than FFQ, the guidelines recommend several (repeated) 24 recalls [55].

However, AHEI was significantly associated with cardiometabolic risk factors, including anthropometric measurements (BMI, WC, and WHR), apo-A, and vessel-related functions (DBP, CDBP, and vascular age). Lavigne-Robichaud et al. [56] compared AHEI with Food Quality Score (FQS) and index, examining the contribution of ultra-processed products (UPP) to total daily dietary energy intake. While all three indices were related to cardiometabolic risk, only the UPP was significantly associated with metabolic syndrome risk [56]. Such rather novel indices could be of interest, as indeed processing techniques and especially ultra-processed food items have been associated with high a intake of sodium, saturated fats, and simple sugars, all of which have been associated with cardiometabolic risk factors [57]. AHEI would also capture similar aspects, as it includes the consumption of fruits and the quality of the consumed fats, though less specifically focusing on sodium and simple sugar intake. In the present study, median AHEI results of 37 (ideal score 75) suggested rather a deviation from the recommended dietary guidelines.

The NNRS, focusing on essential nutrient requirements, showed, as with other nutrient-focused indices, a relatively low association with the observed serum and metabolic parameters. Besides anthropometric markers, fasting blood glucose and sodium (in urine and serum) were significantly associated, which is interesting as neither sodium nor sugars are incorporated into the index. It is possible that factors such as potassium intake played a role (as a high potassium status could reduce sodium re-uptake by the kidneys [58] or that the consumption of proteins was related to lower simple sugar intake [59]. In a study by Kramer et al. on the European elderly, linear regression models analyzed the association between an adapted NNRS and the micronutrient status of folate, vitamin D, vitamin B12, homocysteine, and CRP [60]; a one-unit increase in the adapted NNRS score was associated with a 1.6%/2.2% increase in serum folate for Polish/Dutch participants. The authors also reported a significant inverse association between their NNRS and circulating homocysteine levels, a marker often associated with CVD, in both populations [60]. However, they failed to find a significant association between their NNRS and CRP and serum vitamin D levels [60]. These results are in line with ours, as we also could not find a significant association between NNRS and hs-CRP and serum vitamin D, despite vitamin D intake being part of the NNRS. Results of the NNRS index (median 129%) proposed that the population in Luxembourg did not have any significant deficits in the captured nutrients.

The MDS is another prevalent food-group-based index, which resulted in an intermediate number of significant associations with serum and metabolic parameters. It has been reported [22] that the MDS has a high aptness to predict changes in risk biomarkers and is significantly associated with lower levels of blood pressure, apo-B, renal function indicators (creatinine), and liver enzymes (serum glutamate-pyruvate transaminase and γ-glutamyl-transpeptidase) [22]. These results were similar to our study, revealing significant associations between MDS and BMI, waist circumference, apo-A, urinary creatinine, TSH, and several blood pressure measures. Our study's findings confirm earlier ones [22] and highlight the possibility that a Mediterranean diet can reduce some nutrition-related disease risks. Compared to these earlier reported values [22], also derived from Luxembourg, our present values (median 4) indicate a deviation from the recommendations (ideal score 9), in line with a more Western-type diet, as also emphasized recently [25].

The DAI is a rather recently developed index focusing on a few antioxidants, including vitamins and minerals, as part of antioxidant enzymes. Dietary compounds that could influence antioxidant status via, e.g., transcription factors, e.g., carotenoids or polyphenols, acting on Nrf2 or NF-kB, are not included, even though these factors may play a more significant role in oxidative stress status than direct quenching effects [61]. We reported previously [62], in the Iranian population, a significant association between the DAI and some inflammatory and stress oxidative biomarkers, such as Il-6, MDA, serum insulin, and HOMA-IR [62]. Similarly, in the present study, we also found a significant association between the DAI and some biomarkers, such as hs-CRP, HbA1c, and FBG. However, one of the limitations of the present study is that we did not measure oxidative stress-related biomarkers.

An index that has recently attracted much attention is the DII, due to its relation to a large number of chronic diseases, from cardiometabolic ones [1, 17] to cancer [18], NAFLD [19], and obesity [20]. However, in the present study, this index produced the lowest number of significant associations – only 25-hydroxyvitamin D and urinary creatinine. Several studies have addressed the validation of the DII (a (non-)nutrient-based index) by correlating it with inflammatory markers such as hs-CRP, TNF-a, and IL-6 [18, 63]. However, the only inflammatory marker measured in our study was hs-CRP, and we could not find a significant association between it and the DII. We also examined the association of DII with the SII (previously associated with chronic inflammation [43]), but we failed to find a significant association. However, and interestingly, when we applied an energy-adjusted DII (E-DII, data not shown), a robust and significant association between it and SII was seen in crude and adjusted models (Supplementary Table 6); although the association between E-DII and the hs-CRP still was not significant. It is possible that further adjustment for energy, which is often not included in the indices, would improve the strengths of associations, as, e.g., higher intakes of unhealthy items may merely signal higher energy needs and larger intake, and not necessarily an unhealthier diet.

Correlating indices with each other, we observed that the highest correlations (strong and significant) were between the DAI and NNRS and between the DII and DAI. Given that all of these rather nutrient-based indices, with some overlaps in their considered nutrients, this may not be too surprising. DII and DAI comprise a number of antioxidants, and the NNRS likewise includes several of the same nutrients, emphasizing some redundancy between these. On the other hand, low correlations were obtained for other indices, perhaps pointing to a rather complementarity of these indices, such as between the NNRS (a nutrient-based score) and the DASH-S, MDS, and DQI-I, being rather food-group-based indices. NNRS, for instance, was the single indicator being significantly associated with serum sodium and fasting blood glucose; thus, measuring more than 1 established index may yield further insights into dietary patterns. When investigating meaningful combinations of 2 indices (Supplementary Table 9), the DASH-S diet with either the NNRS or the DAI (both nutrient based or with the AHEI produced the most significant associations with the analyzed parameters (17), highlighting the usefulness of to study combinations of certain, possibly somewhat complementary, indices.

Finally, a critical evaluation of current scoring systems/algorithms for using a priori diet quality scores for CVD risk summarized strengths and limitations of these dietary indices/scores and described index components, calculation methods, and the application of these indices to different population groups [64]. Similar as to our conclusions, the authors emphasized that future applications and interpretations of dietary indices/scores in nutritional epidemiologic studies assessing diet quality should consider food items as well as nutrients when interpreting a score. For instance, scores/indices relying solely on food groups may overlook the importance of the intercorrelation of nutrients with outcomes [64]. It was further suggested that future investigations should consider cross-cultural and other differences between population groups, address the limitations, and identify translational challenges inherent to attempt creating a relevant Diet Quality Index for application in disease prevention at a population level [64].

Our study has several strengths and weaknesses. Examining seven indicators (nutrient, food, and nutrient-food-based indices) for assessing diet quality and associating them with various serum and metabolic biomarkers, and considering typical confounders, was one of the strengths of our study. The measured dietary intake was further derived from an extensive and validated FFQ applied by a trained nurse. This comprehensive contemplation of diet quality using different indicators allowed us to examine multiple aspects of the diet and to emphasize the usefulness of the indices with regard to the observed parameters. However, a limited number of markers for stress oxidative and inflammation were one of the limitations of our study, and though alternatives were investigated, such as the SII as a marker of inflammation, the original DII was not validated against this marker. Another limitation of our study was that it was a cross-sectional survey, so we could not assess the reliability of the indices. Cohort studies with prospective designs would be more suitable to determine the causal relationship between indices and biomarkers and examine their reliability.

## Conclusion

In this study, we examined the association between seven dietary quality indices and serum and metabolic biomarkers in a general adult population. In line with the literature, e.g., in a meta-analysis [65], as opposed to food-based indicators, nutrient-based indices such as the DII and the DAI were less potent than food-group-based indicators such as the DASH-S, DQI-I, or AHEI to predict more general serum indicators and metabolic biomarkers in general populations. Though nutrient-based indicators such as the DII and the DAI have their importance when focusing on more specific populations, due to their higher disease-specificity, for a more general population to reflect less specific cardiometabolic markers and markers of nutrient status such as the ones employed, a rather food-group-based indicator may be considered a more suitable approach. Nevertheless, a combination of complementary indices, such as a general, rather food-group-based one and a more specific, nutrient-based one, is expected to yield more insightful information into a dietary pattern than only a single index would allow. Hence, depending on the targeted health/research question, a combination of carefully selected and complementary indices is advised.

## Supplementary Information

Below is the link to the electronic supplementary material.

Supplementary file1 (DOCX 95 KB)

## Data Availability

Available on request from the corresponding author. Due to our institute's rules and laws, the data are not publicly available.
