# Supplementary material for: Association of diet quality indices with serum and metabolic biomarkers in participants of the ORISCAV-LUX-2 study
Source: Eur J Nutr. 2023 Mar 14;62(5):2063–85. doi: 10.1007/s00394-023-03095-y (PMC10349755; doi:10.1007/s00394-023-03095-y)
Supplement: Supplementary file 1 — Supplementary file1 (DOCX 95 KB) [file 394_2023_3095_MOESM1_ESM.docx]

| **Supplementary Table 1.** Scoring algorithms investigated in the present article. | |
| --- | --- |
| **AHEI [1]** | Principles and criteria for scoring the AHEI included: higher scores were given for a greater intake of vegetables (10 points for 5 servings/day; 0 points for no servings/day) and fruits (10 points for 4 servings/day; 0 points for no servings/day). The ratio of white to red meat was intended to capture a replacement of white for red meat (10 points for a 4:1 ratio; 0 points for '0'– except for vegetarians who received a score of 10). It also included a separate component for non-meat protein sources, including nuts and soy products (10 points for 1 serving/day; 0 points for no servings/day). To capture a higher intake of whole grains, we gave credit for higher cereal fiber intake (10 points for >15 g/day; 0 points for zero g/day). The polyunsaturated to saturated fat ratio was calculated to capture higher consumption of beneficial unsaturated oils (10 points for a ratio ≥1; none for ˂0.1). Moderate alcohol consumption contributed to higher points (10 points for 1.5-2.5 servings/day for men and 0.5-1.5 servings/day for women; 0 points for either no consumption or ˃3.5 servings/day for men and ˃2.5 servings/day women). The multivitamin component was dichotomous, contributing to either 5 points (consuming any supplement) or zero (for all others) to avoid over-weighting this component. For all components, intermediate intakes were scored proportionately between 0 and 10 **(Supplementary Table 2).** |
| **MDS [2]** | Participants whose intake of presupposed advantageous items (fish, legumes, vegetables, fruits, and grains) was below the median intake were assigned a 0 and a point of 1 otherwise. Participants whose intake of presupposed adverse items (dairy products and meat) was below the median intake were assigned a point of 1 and 0 otherwise. A point of 1 was given to men consuming from 10 grams to less than 50 grams of ethanol (alcohol) per day and women from 5 to 25 g/day. The ratio of MUFAs to SFAs was computed for lipid consumption, a ratio below the median were assigned a 0 and a point of 1 otherwise. |
| **DASH [3]** | The component score for fruits, vegetables, nuts and legumes, dairy products, and whole grains is the participant’s quintile ranking. For example, quintile 1 was assigned 1 point and the fifth quintile 5 points. Low intake was desired for sodium, red and processed meats, and sweetened beverages. Therefore, the lowest quintile scored 5 points and the highest quintile 1 point **(Supplementary Table 3).** |
| **DQI-I [4]** | The four major categories **(Supplementary Table 4**) of this index were:  **Variety**. Including at least one serving of food per day from each of the five food groups (meat/poultry/fish/egg, dairy/beans, grains, fruits, and vegetables) defined the maximum overall variety score. If intake of any of these food groups was missing, the score was reduced from the maximum score of 15 by 3 points each per food group. **Adequacy**. The scores for the eight components in the category were assigned based on the percentage attainment of the recommended intakes on a continuous scale, which ranged from 0 points for 0% to 5 points for 100%, with a cap at 5 points. A diet that contained ≥2 to 4 servings of fruits and ≥3 to 5 servings of vegetables, depending on three levels of energy intake (1700, 2200, and 2700 kcal] was given the highest score of 5 points. Daily consumptions of ≥6, 9, and 11 servings from the grain group and 20, 25, and 30 grams of fiber for the 3 energy consumption categories, respectively, met the criteria for the highest score for the grain and fiber components. Consumption of protein was considered adequate when the proportion of total energy intake from protein is 10%. The level of intake that defined the highest score for adequacy of iron, calcium, and vitamin C was derived from the DRI, which varied by gender and age. **Moderation**. The intake levels of these nutrients were categorized into three tiers according to the degree of effect on health. The lowest intake category was given the highest score of 6 points, the highest intake category the lowest score of 0, and the middle tier a score of 3 points. When energy from total fat was ≤20% of total energy, the highest score was given; when ˃30%, the lowest score is given. SFA intake was also evaluated based on the percentage of energy from SFA. Intakes of cholesterol and sodium were examined based on the intake level. One of the unique components of the DQI-I was evaluating the intake of low nutrient density foods, the so-called "empty calorie foods." This component assesses how much of a person's energy supply depends on low nutrient density foods, which provided only energy but insufficient nutrients. Foods such as table sugar, oil, and alcohol were examples of empty-calorie foods. In the DQI-I, if the sum of nutrient densities across nutrients examined in food was ˂1, the food was considered an empty-calorie food. The lowest score was assigned when the energy supplied by empty-calorie foods was ˃10% of total energy intake per day. **Overall balance**. The final category examined the overall balance of diet proportionality in energy sources and fatty acid composition. Proportionality in energy sources and fatty acid composition each contributed to the total DQI-I by 6 and 4 points, respectively. |
| **DII [5]** | Calculation of the DII was based on dietary intake data linked to a regionally representative world database that provided a robust estimate of a mean and standard deviation for each parameter. These then became the multipliers to express an individual's exposure relative to the 'standard global mean' as a Z-score. This was achieved by subtracting the 'standard mean' from the reported amount and dividing this value by its standard deviation (means and standard deviations for all 45 parameters are shown in **Supplementary Table 5).** |
| **DAI [6]** | Intake of each antioxidant nutrient was standardized by subtracting the mean and dividing it by the standard deviation. For a given set of nutrients, the resulting principal components represented linear combinations of the original variables. In other words, each principal component was computed by first multiplying the standardized intake of a specific nutrient by its corresponding weight or factor loading and subsequently summing up across all contributing nutrients to obtain a score for each study participant. A summation of the principal component scores, plus selenium and vitamin C, was carried out to derive the composite antioxidant index. The principal components and nutrients (selenium, vitamin C) were equally weighted in the summed index. The index was subsequently adjusted for energy via the residual method. |
| **NNRS [7]** | The NNRS is the average of %DVs for 14 key nutrients:  NNR=∑%DV2000 kcal/14; NNRS for each nutrient= ((DV/2000)/(intake/energy intake)) * 100  Recommended daily values (DVs) based on the dietary reference intakes: Protein 65 g; Vitamin A 5000 IU; Vitamin C 75 mg; Calcium 1300 mg; Iron 18 mg; Zinc 11 mg; Folate 400 μg; Thiamine 1.2 mg; Riboflavin 1.3 mg; Vitamin B-12 2.4 μg; Vitamin D 10 μg; Vitamin E 15 mg; MUFA 20 g; Potassium 3500 mg. |

| **Supplementary Table 2.** Alternative Healthy Eating Index (AHEI) scoring algorithm [1]. | | | |
| --- | --- | --- | --- |
| Component | Criteria for minimum score of 0^*^ | Criteria for maximum score of 0^*^ | Remarks |
| Vegetables (servings/day) | 0 | 5 | All vegetables on the FFQ were included, except potatoes (including French fries), because they have a high glycemic index. Five servings of vegetables/day were considered ideal, reflecting the upper range of current dietary guidelines. |
| Fruit (servings/day) | 0 | 4 | Four servings/day was considered ideal, consistent with the upper range of current dietary guidelines. |
| Nuts and soy protein (servings/day) | 0 | 1 | A combined average of 1 serving/day was considered ideal. |
| Ratio of white to red meat | 0 | 4 | White meat was defined as poultry or fish, whereas beef, pork, lamb, and processed meats were considered red meat. Gram quantities were summed and used in calculating the ratio. An ideal score of 10 was given for ratios ≥4:1. This value was chosen arbitrarily but is practical and consistent with patterns in healthy populations. Vegetarians and those who were consuming red meat <2 times/month were given a score of 10. |
| Cereal fiber (g/day) | 0 | 15 | 15 g cereal fiber/day was considered ideal based on epidemiologic studies. |
| Trans-fatty acids | ≥4 | ≤0.5 | A trans fatty acid intake of ≤0.5% of total energy was considered ideal, whereas ≥4% was assigned the lowest score. |
| P:S | ≤0.1 | ≥1 | A P:S ≥1 was considered to be ideal. A ratio of ≤0.1 was the least optimal. |
| Duration of multivitamin use ^a^ | <5 years | ≥5 years | This is the only variable of the index that is not continuous. To avoid overweighting the score from this component, participants who had used multivitamins for ≥5 y scored 7.5 (best); for all others, a score of 2.5 was given. |
| Alcohol (servings/day)^b^ | Men: 0 or >3.5  Women: 0 or >2.5 | Men: 1.5-2.5  Women: 0.5-1.5 | Moderate alcohol consumption of 1.5-2.5 drinks/day was defined as ideal for men, and 0.5-1.5 drinks/d as ideal for women. |
| Total score (range) | 2.5 | 87.5 |  |
| FFQ, food-frequency questionnaire; P:S, a polyunsaturated to saturated fatty acids ratio.  * Intermediate intakes are scored proportionately between 0 and 10.  ^a^ For multivitamins, the minimum score is 2.5, and the maximum score is 7.5.  ^b^ Beer, wine, and liquor. | | | |

| **Supplementary Table 3.** Scoring criteria for the DASH-style diet [3]. | | |
| --- | --- | --- |
| Component | Foods | Scoring Criteria |
| Fruits | All fruits and fruit juices | Q1 = 1 point  Q2 = 2 points  Q3 = 3 points  Q4 = 4 points  Q5 = 5 points |
| Vegetables | All vegetables except potatoes and legumes |  |
| Nuts and legumes | Nuts and peanut butter, dried beans, peas, tofu |  |
| Whole grains | Brown rice, dark bread, cooked cereal, whole-grain cereal,  Other grains, popcorn, wheat germ, bran |  |
| Low-fat dairy | Skim milk, yogurt, cottage cheese |  |
| Sodium * | Sum of the sodium content of all foods in FFQ | Reverse scoring:  Q1 = 5 points  Q2 = 4 points  Q3 = 3 points  Q4 = 4 points  Q5 = 1 point |
| Red and processed meats * | Beef, pork, lamb, deli meats, organ meats, hot dogs, bacon |  |
| Sweetened beverages * | Carbonated and noncarbonated sweetened beverages |  |
| * Higher quintiles represent higher intake; however, high intake and high quintiles received lower scores in constructing the DASH score. | | |

| **Supplementary Table 4.** DQI-I scoring system [4]. | | |
| --- | --- | --- |
| Component | Score | Scoring criteria |
| **Variety** | 0–20 points |  |
| Overall food group variety (meat/poultry/fish/eggs; dairy/beans; grain; fruit; vegetable) | 0–15 points | ≥1 serving from each food group/d = 15 |
|  |  | Any 1 food group missing/d = 12 |
|  |  | Any 2 food groups missing/d = 9 |
|  |  | Any 3 food groups missing/d = 6 |
|  |  | ≥4 food groups missing/d = 3 |
|  |  | None from any food groups = 0 |
| Within-group variety for protein source (meat, poultry, fish, dairy, beans, eggs) | 0–5 points | ≥3 different sources/d = 5 |
|  |  | 2 different sources/d = 3 |
|  |  | From 1 source/d = 1 |
|  |  | None = 0 |
| **Adequacy** | 0–40 points |  |
| Vegetable group^1, 2^ | 0–5 points | ≥3–5 servings/d = 5, 0 servings/d = 0 |
|  |  | ≥100% |
|  |  | <100–50% |
|  |  | <50% |
| Fruit group^1, 2^ | 0–5 points | ≥2–4 servings/d = 5, 0 servings/d = 0 |
|  |  | ≥100% |
|  |  | <100–50% |
|  |  | <50% |
| Grain group^1, 2^ | 0–5 points | ≥6–11 servings/d = 5, 0 servings/d = 0 |
|  |  | ≥100% |
|  |  | <100–50% |
|  |  | <50% |
| Fiber^1, 2^ | 0–5 points | ≥20–30 g/d = 5, 0 g/d = 0 |
|  |  | ≥100% |
|  |  | <100–50% |
|  |  | <50% |
| Protein^1^ | 0–5 points | ≥10% of energy/d = 5, 0% of energy/d = 0 |
|  |  | ≥100% |
|  |  | <100–50% |
|  |  | <50% |
| Iron^1, 3^ | 0–5 points | ≥100% RDA (AI)/d = 5, 0% RDA (AI)/d = 0 |
|  |  | ≥100% |
|  |  | <100–50% |
|  |  | <50% |
| Calcium^1^ | 0–5 points | ≥100% AI/d = 5, 0% AI/d = 0 |
|  |  | ≥100% |
|  |  | <100–50% |
|  |  | <50% |
| Vitamin C^1, 4^ | 0–5 points | ≥100% RDA (RNI)/d = 5, 0% RDA (RNI)/d = 0 |
|  |  | ≥100% |
|  |  | <100–50% |
|  |  | <50% |
| **Moderation** | 0–30 points |  |
| Total fat | 0–6 points | ≤20% of total energy/d = 6 |
|  |  | >20–30% of total energy/d = 3 |
|  |  | >30% of total energy/d = 0 |
| Saturated fat | 0–6 points | ≤7% of total energy/d = 6 |
|  |  | >7–10% of total energy/d = 3 |
|  |  | >10% of total energy/d = 0 |
| Cholesterol | 0–6 points | ≤300 mg/d = 6 |
|  |  | >300–400 mg/d = 3 |
|  |  | >400 mg/d = 0 |
| Sodium | 0–6 points | ≤2400 mg/d = 6 |
|  |  | >2400–3400 mg/d = 3 |
|  |  | >3400 mg/d = 0 |
| Empty calorie foods | 0–6 points | ≤3% of total energy/d = 6 |
|  |  | >3–10% of total energy/d = 3 |
|  |  | >10% of total energy/d = 0 |
| **Overall balance** | 0–10 points |  |
| Macronutrient ratio^5^ (carbohydrate:protein: fat) | 0–6 points | 55 ∼ 65:10 ∼ 15:15 ∼ 25 = 6 |
|  |  | 52 ∼ 68:9 ∼ 16:13 ∼ 27 = 4 |
|  |  | 50 ∼ 70:8 ∼ 17:12 ∼ 30 = 2 |
|  |  | Otherwise = 0 |
| Fatty acid ratio (PUFA:MUFA: SFA) | 0–4 points | P/S = 1 ∼ 1.5 and M/S = 1 ∼ 1.5 = 4 |
|  |  | Else if P/S = 0.8 ∼ 1.7 and M/S = 0.8 ∼ 1.7 = 2 |
|  |  | Otherwise = 0 |
| ^1^ Used as a continuous variable. ^2^ Based on 7118 kJ (1700 kcal)/9211 kJ (2200 kcal)/11304 kJ (2700 kcal) diet; 1 kcal = 4.1868 kJ. ^3^ Scoring system based on the Adequate Intake (AI) and Dietary Reference Intakes (RDA) values. ^4^ Scoring system based on the Reference Nutrient Intake (RNI) and RDA values. ^5^ Ratio of energy from carbohydrate to protein to fat. | | |

| **Supplementary Table 5.** Food parameters included in the dietary inflammatory index, inflammatory effect scores, and intake values from the global composite data set [5]. | | | | |
| --- | --- | --- | --- | --- |
| Food parameter | Raw inflammatory effect score* | Overall inflammatory effect score^†^ | Global daily mean intake (unit/day) ^‡^ | SD^‡^ |
| Alcohol (g) | –0·278 | –0·278 | 13·98 | 3·72 |
| Vitamin B12 (µg) | 0·205 | 0·106 | 5·15 | 2·70 |
| Vitamin B6 (mg) | –0·379 | –0·365 | 1·47 | 0·74 |
| β-Carotene (µg) | –0·584 | –0·584 | 3718 | 1720 |
| Caffeine (mg) | –0·124 | –0·110 | 8·05 | 6·67 |
| Carbohydrate (g) | 0·109 | 0·097 | 272·2 | 40·0 |
| Cholesterol (mg) | 0·347 | 0·110 | 279·4 | 51·2 |
| Energy (kcal) | 0·180 | 0·180 | 2056 | 338 |
| Eugenol (mg) | –0·868 | –0·140 | 0·01 | 0·08 |
| Total fat (g) | 0·298 | 0·298 | 71·4 | 19·4 |
| Fiber (g) | –0·663 | –0·663 | 18·8 | 4·9 |
| Folic acid (µg) | –0·207 | –0·190 | 273·0 | 70·7 |
| Garlic (g) | –0·412 | –0·412 | 4·35 | 2·90 |
| Ginger (g) | –0·588 | –0·453 | 59·0 | 63·2 |
| Fe (mg) | 0–032 | 0·032 | 13·35 | 3·71 |
| Mg (mg) | –0·484 | –0·484 | 310·1 | 139·4 |
| MUFA (g) | –0·019 | –0·009 | 27·0 | 6·1 |
| Niacin (mg) | –1·000 | –0·246 | 25·90 | 11·77 |
| n-3 Fatty acids (g) | –0·436 | –0·436 | 1·06 | 1·06 |
| n-6 Fatty acids (g) | –0·159 | –0·159 | 10·80 | 7·50 |
| Onion (g) | –0·490 | –0·301 | 35·9 | 18·4 |
| Protein (g) | 0·049 | 0·021 | 79·4 | 13·9 |
| PUFA (g) | –0·337 | –0·337 | 13·88 | 3·76 |
| Riboflavin (mg) | –0·727 | –0·068 | 1·70 | 0·79 |
| Saffron (g) | –1·000 | –0·140 | 0·37 | 1·78 |
| Saturated fat (g) | 0·429 | 0·373 | 28·6 | 8·0 |
| Se (µg) | –0·191 | –0·191 | 67·0 | 25·1 |
| Thiamine (mg) | –0·354 | –0·098 | 1·70 | 0·66 |
| Trans fat (g) | 0·432 | 0·229 | 3·15 | 3·75 |
| Turmeric (mg) | –0·785 | –0·785 | 533·6 | 754·3 |
| Vitamin A (RE) | –0·401 | –0·401 | 983·9 | 518·6 |
| Vitamin C (mg) | –0·424 | –0·424 | 118·2 | 43·46 |
| Vitamin D (µg) | –0·446 | –0·446 | 6·26 | 2·21 |
| Vitamin E (mg) | –0·419 | –0·419 | 8·73 | 1·49 |
| Zn (mg) | –0·313 | –0·313 | 9·84 | 2·19 |
| Green/black tea (g) | –0·536 | –0·536 | 1·69 | 1·53 |
| Flavan-3-ol (mg) | –0·415 | –0·415 | 95·8 | 85·9 |
| Flavones (mg) | –0·616 | –0·616 | 1·55 | 0·07 |
| Flavonols (mg) | –0·467 | –0·467 | 17·70 | 6·79 |
| Flavanones (mg) | –0·908 | –0·250 | 11·70 | 3·82 |
| Anthocyanidins (mg) | –0·449 | –0·131 | 18·05 | 21·14 |
| Isoflavones (mg) | –0·593 | –0·593 | 1·20 | 0·20 |
| Pepper (g) | –0·397 | –0·131 | 10·00 | 7·07 |
| Thyme/oregano (mg) | –1·000 | –0·102 | 0·33 | 0·99 |
| Rosemary (mg) | –0·333 | –0·013 | 1·00 | 15·00 |
| * This is referred to as the 'food parameter-specific raw inflammatory effect score' in the text and is abbreviated here for ease of presentation. Note that the effect is per unit amount noted for each food parameter. ^†^ This refers to the 'food parameter-specific overall inflammatory effect score' accounting for the robustness of the literature. ^‡^ From the world composite database. | | | | |

| **Supplementary Table 6.** Linear regression^a^ of the associations between diet quality indices (continuous) and serum and metabolic biomarkers. | | | | | | | | | | | | | | | | | | | | | | | | | | | | | | | | | | | | | |
| --- | --- | --- | --- | --- | --- | --- | --- | --- | --- | --- | --- | --- | --- | --- | --- | --- | --- | --- | --- | --- | --- | --- | --- | --- | --- | --- | --- | --- | --- | --- | --- | --- | --- | --- | --- | --- | --- |
|  | | | AHEI | | | | | MDS | | | | | | DASH-S | | | | | | DQI-I | | | | | DII | | | | | DAI | | | | NNRS | | | |
|  |  |  | β (95%CI) | | P-value | | | β (95%CI) | | | P-value | | | β (95%CI) | | | P-value | | | β (95%CI) | | | P-value | | β (95%CI) | | | P-value | | β (95%CI) | | P-value | | β (95%CI) | | P-value | |
| **Anthropometry** | | | | | | | | | | | | | | | | | | | | | | | | | | | | | | | | | | | | | |
| BMI (kg/m^2^) | | | -0.028 (-0.048, -0.007) | | 0.008 | | | -0.247 (-0.399, -0.095) | | | 0.001 | | | -0.135 (-0.189, -0.081) | | | <0.001 | | | -0.051 (-0.082, -0.019) | | | 0.002 | | -0.065 (-0.195, 0.065) | | | 0.326 | | 0.092 (0.052, 0.132) | | <0.001 | | 0.012 (0.007, 0.017) | | <0.001 | |
| WC (cm) | | | -0.053 (-0.111, 0.005) | | 0.073 | | | -0.859 (-1.288, -0.430) | | | <0.001 | | | -0.439 (-0.592, -0.285) | | | <0.001 | | | -0.177 (-0.265, -0.088) | | | <0.001 | | -0.557 (-0.924, -0.191) | | | 0.003 | | 0.400 (0.288, 0.511) | | <0.001 | | 0.054 (0.039, 0.069) | | <0.001 | |
| WHR | | | -0.000 (-0.001, 0.000) | | 0.080 | | | -0.006 (-0.009, -0.003) | | | <0.001 | | | -0.003 (-0.004, -0.002) | | | <0.001 | | | -0.001 (-0.002, -0.001) | | | <0.001 | | -0.003 (-0.006, -0.001) | | | 0.015 | | 0.003 (0.002, 0.003) | | <0.001 | | 0.000 (0.000, 0.000) | | <0.001 | |
| **Inflammation related measurements** | | | | | | | | | | | | | | | | | | | | | | | | | | | | | | | | | | | | | |
| hsCRP (μg/L) | | | -0.018 (-0.042, 0.006) | | 0.135 | | | -0.044 (-0.220, 0.132) | | | 0.625 | | | -0.024 (-0.088, 0.039) | | | 0.455 | | | -0.000 (-0.037, 0.036) | | | 0.996 | | 0.106 (-0.045, 0.256) | | | 0.168 | | 0.001 (-0.045, 0.048) | | 0.966 | | -0.001 (-0.007, 0.006) | | 0.866 | |
| SII | | | -0.333 (-1.325, 0.658) | | 0.510 | | | -4.320 (-11.675, 3.034) | | | 0.249 | | | -0.906 (-3.565, 1.753) | | | 0.504 | | | -0.467 (-1.987, 1.053) | | | 0.547 | | 1.027 (-5.263, 7.316)* | | | 0.749 | | -0.348 (-2.289, 1.593) | | 0.725 | | 0.005 (-0.257, 0.266) | | 0.973 | |
| **Glucose related measurements** | | | | | | | | | | | | | | | | | | | | | | | | | | | | | | | | | | | | | |
| Insulin (μg/L) | | | -0.044 (-0.097, 0.009) | | 0.106 | | | -0.236 (-0.629, 0.157) | | | 0.240 | | | 0.022 (-0.120, 0.165) | | | 0.758 | | | -0.018 (-0.099, 0.063) | | | 0.665 | | 0.161 (-0.174, 0.496) | | | 0.345 | | 0.053 (-0.051, 0.157) | | 0.316 | | 0.006 (-0.008, 0.020) | | 0.421 | |
| HOMA-IR | | | -0.349 (-0.760, 0.062) | | 0.096 | | | -1.932 (-4.986, 1.123) | | | 0.215 | | | 0.297 (-0.811, 1.406) | | | 0.599 | | | -0.135 (-0.769, 0.499) | | | 0.676 | | 1.509 (-1.099, 4.116) | | | 0.257 | | 0.230 (-0.584, 1.044) | | 0.579 | | 0.013 (-0.096, 0.123) | | 0.810 | |
| HbA1c (%) | | | 0.012 (-0.011, 0.035) | | 0.315 | | | 0.025 (-0.149, 0.198) | | | 0.782 | | | 0.080 (0.018, 0.143) | | | 0.012 | | | 0.047 (0.011, 0.083) | | | 0.011 | | -0.048 (-0.196, 0.101) | | | 0.529 | | 0.043 (-0.002, 0.089) | | 0.064 | | 0.004 (-0.002, 0.011) | | 0.170 | |
| FBS (mg/dL) | | | -0.044 (-0.120, 0.032) | | 0.257 | | | -0.796 (-1.359, -0.234) | | | 0.006 | | | -0.208 (-0.411, -0.005) | | | 0.045 | | | -0.071 (-0.188, 0.046) | | | 0.233 | | -0.122 (-0.603, 0.359) | | | 0.619 | | 0.209 (0.061, 0.357) | | 0.006 | | 0.026 (0.006, 0.046) | | 0.011 | |
| **Lipid related measurements** | | | | | | | | | | | | | | | | | | | | | | | | | | | | | | | | | | | | | |
| Apo A (mg/L) | | | -0.077 (-0.201, 0.047) | | 0.222 | | | -0.314 (-1.236, 0.609) | | | 0.505 | | | 0.450 (0.117, 0.782) | | | 0.008 | | | -0.027 (-0.217, 0.164) | | | 0.783 | | 0.833 (0.047, 1.618) | | | 0.038 | | -0.607 (-0.848, -0.366) | | <0.001 | | -0.086 (-0.118, -0.053) | | <0.001 | |
| Apo B (mg/L) | | | -0.054 (-0.151, 0.044) | | 0.278 | | | -0.495 (-1.219, 0.229) | | | 0.180 | | | -0.496 (-0.756, -0.235) | | | <0.001 | | | -0.228 (-0.377, -0.079) | | | 0.003 | | -0.594 (-1.211, 0.023) | | | 0.059 | | 0.287 (0.096, 0.477) | | 0.003 | | 0.040 (0.015, 0.066) | | 0.002 | |
| TG (mg/dL) | | | -0.532 (-0.934, -0.131) | | 0.009 | | | -3.740 (-6.720, -0.761) | | | 0.014 | | | -2.286 (-3.358, -1.214) | | | <0.001 | | | -0.986 (-1.601, -0.371) | | | 0.002 | | 1.065 (-1.483, 3.613) | | | 0.436 | | 0.483 (-0.305, 1.271) | | 0.230 | | 0.081 (-0.025, 0.187) | | 0.133 | |
| Total cholesterol (mg/dL) | | | -0.056 (-0.223, 0.111) | | 0.510 | | | -0.399 (-1.638, 0.917) | | | 0.528 | | | -0.271 (-0.719, 0.176) | | | 0.235 | | | -0.275 (-0.531, -0.019) | | | 0.035 | | -0.761 (-1.818, 0.297) | | | 0.158 | | 0.123 (-0.204, 0.450) | | 0.461 | | 0.019 (-0.025, 0.063) | | 0.400 | |
| LDL (mg/dL) | | | 0.004 (-0.143, 0.152) | | 0.953 | | | -0.005 (-1.099, 1.089) | | | 0.993 | | | -0.343 (-0.738, 0.052) | | | 0.089 | | | -0.199 (-0.425, 0.027) | | | 0.084 | | -1.007 (-1.940, -0.075) | | | 0.034 | | 0.303 (0.014, 0.591) | | 0.040 | | 0.043 (0.004, 0.082) | | 0.031 | |
| HDL (mg/dL) | | | 0.036 (-0.027, 0.099) | | 0.262 | | | 0.370 (-0.099, 0.839) | | | 0.122 | | | 0.467 (0.300, 0.635) | | | <0.001 | | | 0.103 (0.006, 0.200) | | | 0.037 | | 0.146 (-0.255, 0.547) | | | 0.477 | | -0.298 (-0.421, -0.174) | | <0.001 | | -0.042 (-0.059, -0.025) | | <0.001 | |
| **Kidney related measurement** | | | | | | | | | | | | | | | | | | | | | | | | | | | | | | | | | | | | | |
| Urinary microalbumin (mg/L) | | | -0.183 (-0.437, 0.070) | | 0.156 | | | -1.187 (-3.054, 0.680) | | | 0.212 | | | -0.027 (-0.704, 0.649) | | | 0.937 | | | -0.155 (-0.541, 0.231) | | | 0.431 | | 1.521 (-0.083, 3.124) | | | 0.063 | | -0.217 (-0.714, 0.280) | | 0.391 | | -0.030 (-0.097, 0.037) | | 0.381 | |
| Urinary creatinine (μM) | | | -1.089 (-1.422, -0.755) | | <0.001 | | | -7.903 (-10.364, -5.442) | | | <0.001 | | | -3.533 (-4.418, -2.649) | | | <0.001 | | | -1.997 (-2.502, -1.492) | | | <0.001 | | 2.266 (0.122, 4.411) | | | 0.038 | | 0.284 (-0.380, 0.949) | | 0.401 | | 0.084 (-0.005, 0.174) | | 0.065 | |
| Albumin/creatinine | | | -0.188 (-0.536, 0.160) | | 0.289 | | | -1.090 (-3.595, 1.415) | | | 0.393 | | | 0.113 (-0.806, 1.033) | | | 0.809 | | | -0.231 (-0.755, 0.292) | | | 0.386 | | 2.295 (0.097, 4.493) | | | 0.041 | | -0.411 (-1.095, 0.273) | | 0.238 | | -0.050 (-0.142, 0.042) | | 0.284 | |
| Uric acid (mg/dL) | | | -0.008 (-0.014, -0.002) | | 0.005 | | | -0.094 (-0.136, -0.052) | | | <0.001 | | | -0.055 (-0.070, -0.039) | | | <0.001 | | | -0.021 (-0.029, -0.012) | | | <0.001 | | -0.035 (-0.071, 0.002) | | | 0.062 | | 0.029 (0.018, 0.040) | | <0.001 | | 0.004 (0.003, 0.006) | | <0.001 | |
| **Nutritional status** | | | | | | | | | | | | | | | | | | | | | | | | | | | | | | | | | | | | | |
| Plasma Vitamin D (ng/mL) | | | 0.138 (0.089, 0.186) | | <0.001 | | | 0.616 (0.253, 0.979) | | | 0.001 | | | 0.378 (0.428, 0.508) | | | <0.001 | | | 0.197 (0.122, 0.271) | | | <0.001 | | -0.324 (-0.634, -0.014) | | | 0.040 | | -0.026 (-0.122, 0.070) | | 0.594 | | -0.009 (-0.022, 0.004) | | 0.182 | |
| Calcium in serum (mg/dL) | | | 0.001 (-0.002, 0.001) | | 0.625 | | | -0.000 (-0.012, 0.011) | | | 0.950 | | | -0.004 (-0.008, 0.001) | | | 0.084 | | | -0.001 (-0.003, 0.002) | | | 0.568 | | -0.001 (-0.011, 0.009) | | | 0.783 | | 0.002 (-0.002, 0.005) | | 0.344 | | -0.000 (-0.000, 0.001) | | 0.680 | |
| Urinary sodium (mg/dL) | | | -0.215 (-0.415, -0.015) | | 0.036 | | | -0.916 (-2.393, 0.561) | | | 0.224 | | | -1.461 (-1.990, -0.932) | | | <0.001 | | | -0.666 (-0.969, -0.363) | | | <0.001 | | 0.362 (-0.909, 1.632) | | | 0.577 | | 0.386 (-0.008, 0.779) | | 0.055 | | 0.076 (0.023, 0.129) | | 0.005 | |
| Sodium in serum (mg/dL) | | | 0.009 (0.001, 0.017) | | 0.027 | | | 0.037 (-0.021, 0.095) | | | 0.215 | | | 0.002 (-0.019, 0.023) | | | 0.861 | | | 0.007 (-0.005, 0.019) | | | 0.265 | | -0.043 (-0.093, 0.006) | | | 0.086 | | 0.022 (0.007, 0.038) | | 0.004 | | 0.003 (0.001, 0.005) | | 0.002 | |
| Potassium serum (mg/dL) | | | -0.000 (-0.001, 0.001) | | 0.918 | | | -0.001 (-0.011, 0.009) | | | 0.804 | | | -0.001 (-0.004, 0.003) | | | 0.631 | | | -0.001 (-0.003, 0.001) | | | 0.205 | | -0.002 (-0.010, 0.007) | | | 0.705 | | 0.001 (-0.002, 0.004) | | 0.432 | | 0.000 (-0.000, 0.001) | | 0.323 | |
| Mg in serum (mg/dL) | | | 0.000 (-0.000, 0.001) | | 0.151 | | | -0.000 (-0.005, 0.005) | | | 0.973 | | | 0.001 (-0.001, 0.003) | | | 0.208 | | | 0.001 (-0.000, 0.002) | | | 0.165 | | -0.001 (-0.005, 0.003) | | | 0.628 | | 0.000 (-0.001, 0.002) | | 0.646 | | -0.000 (-0.000, 0.000) | | 0.863 | |
| Ferritin (ng/mL) | | | -0.721 (-1.325, -0.117) | | 0.019 | | | -6.816 (-11.301, -2.332) | | | 0.003 | | | -5.029 (-6.634, -3.424) | | | <0.001 | | | -2.082 (-3.006, -1.159) | | | <0.001 | | -3.370 (-7.196, 0.456) | | | 0.084 | | 2.284 (1.106, 34.63) | | <0.001 | | 0.344 (0.185, 0.503) | | <0.001 | |
| Haematocrit (%) | | | -0.014 (-0.029, 0.002) | | 0.079 | | | -0.286 (-0.399, -0.173) | | | <0.001 | | | -0.132 (-0.172, -0.091) | | | <0.001 | | | -0.060 (-0.083, -0.036) | | | <0.001 | | -0.115 (-0.212, -0.017) | | | 0.021 | | 0.086 (0.056, 0.116) | | <0.001 | | 0.013 (0.009, 0.017) | | <0.001 | |
| Hemoglobin (g/L) | | | -0.006 (-0.012, -0.001) | | 0.031 | | | -0.117 (-0.158, -0.076) | | | <0.001 | | | -0.050 (-0.065, -0.035) | | | <0.001 | | | -0.024 (-0.033, -0.016) | | | <0.001 | | -0.040 (-0.076, -0.005) | | | 0.026 | | 0.031 (0.021, 0.042) | | <0.001 | | 0.005 (0.003, 0.006) | | <0.001 | |
| **Hormonal status** | | | | | | | | | | | | | | | | | | | | | | | | | | | | | | | | | | | | | |
| TSH (mIU/L) | | | -0.006 (-0.010, -0.001) | | 0.019 | | | -0.035 (-0.069, -0.000) | | | 0.048 | | | -0.010 (-0.023, 0.002) | | | 0.116 | | | -0.005 (-0.012, 0.002) | | | 0.149 | | 0.020 (-0.010, 0.049) | | | 0.192 | | -0.005 (-0.014, 0.004) | | 0.307 | | -0.000 (-0.002, 0.001) | | 0.661 | |
| Free T3 (pmol/L) | | | -0.001 (-0.003, 0.001) | | 0.287 | | | -0.007 (-0.021, 0.007) | | | 0.319 | | | -0.004 (-0.009, 0.001) | | | 0.127 | | | -0.001 (-0.004, 0.002) | | | 0.479 | | -0.001 (-0.013, 0.011) | | | 0.894 | | 0.002 (-0.002, 0.005) | | 0.332 | | 0.000 (-0.000, 0.001) | | 0.482 | |
| Free T4 (ng/dL) | | | 0.001 (-0.001, 0.002) | | 0.437 | | | 0.003 (-0.008, 0.014) | | | 0.606 | | | -0.000 (-0.004, 0.0004) | | | 0.988 | | | -0.000 (-0.002, 0.002) | | | 0.971 | | -0.005 (-0.014, 0.005) | | | 0.316 | | 0.003 (-0.000, 0.006) | | 0.052 | | 0.000 (0.000, 0.001) | | 0.015 | |
| **Vascular function and cardiovascular risk** | | | | | | | | | | | | | | | | | | | | | | | | | | | | | | | | | | | | | |
| SBP (mmHg) | | | -0.032 (-0.105, 0.041) | | 0.385 | | | -1.385 (-1.921, -0.848) | | | <0.001 | | | -0.238 (-0.432, -0.044) | | | 0.016 | | | -0.114 (-0.225, -0.002) | | | 0.046 | | -0.257 (-0.719, 0.204) | | | 0.274 | | 0.127 (-0.015, 0.270) | | 0.079 | | 0.014 (-0.005, 0.034) | | 0.139 | |
| CSBP (mmHg) | | | -0.077 (-0.152, -0.003) | | 0.042 | | | -1.527 (-2.077, -0.976) | | | <0.001 | | | -0.321 (-0.520, -0.122) | | | 0.002 | | | -0.182 (-0.297, -0.067) | | | 0.002 | | -0.018 (-0.493, 0.458) | | | 0.942 | | 0.087 (-0.060, 0.234) | | 0.244 | | 0.012 (-0.008, 0.032) | | 0.244 | |
| DBP (mmHg) | | | -0.069 (-0.115, -0.023) | | 0.003 | | | -0.887 (-1.225, -0.548) | | | <0.001 | | | -0.257 (-0.379, -0.135) | | | <0.001 | | | -0.107 (-0.117, -0.036) | | | 0.003 | | 0.037 (-0.255, 0.328) | | | 0.804 | | 0.028 (-0.062, 0.118) | | 0.538 | | 0.002 (-0.010, 0.014) | | 0.762 | |
| CDBP (mmHg) | | | -0.079 (-0.122, -0.037) | | <0.001 | | | -0.855 (-1.168, -0.542) | | | <0.001 | | | -0.220 (-0.333, -0.107) | | | <0.001 | | | -0.117 (-0.182, -0.051) | | | <0.001 | | 0.142 (-0.128, 0.412) | | | 0.303 | | 0.014 (-0.070, -0.098) | | 0.741 | | -0.000 (-0.012, 0.011) | | 0.966 | |
| GFR^b^ (ml/min/1.73m^2^) | | | -0.003 (-0.060, 0.054) | | 0.912 | | | -0.018 (-0.439, 0.403) | | | 0.933 | | | -0.231 (-0.383, -0.079) | | | 0.003 | | | -0.084 (-0.171, 0.003) | | | 0.059 | | -0.273 (-0.632, 0.085) | | | 0.135 | | 0.090 (-0.021, 0.201) | | 0.113 | | 0.015 (0.000, 0.030) | | 0.045 | |
| PWV (m/s) | | | 0.004 (-0.025, 0.033) | | 0.789 | | | 0.213 (-0.002, 0.427) | | | 0.053 | | | 0.060 (-0.017, 0.138) | | | 0.125 | | | 0.042 (-0.003, 0.086) | | | 0.068 | | 0.045 (-0.139, 0.229) | | | 0.630 | | -0.034 (-0.091, 0.023) | | 0.244 | | -0.006 (-0.013, 0.002) | | 0.139 | |
| Vascular age (y) | | | -0.094 (-0.175, -0.012) | | 0.024 | | | -0.660 (-1.264, -0.056) | | | 0.032 | | | -0.157 (-0.377, 0.062) | | | 0.159 | | | -0.085 (-0.213, 0.042) | | | 0.188 | | 0.467 (-0.054, 0.987) | | | 0.079 | | -0.082 (-0.241, 0.078) | | 0.315 | | -0.014 (-0.035, 0.008) | | 0.203 | |
| ^a^Crude models; ^b^Estimated by Modification of Diet in Renal Disease (MDRD) method.  AHEI= Alternative Healthy Eating Index, MDS= Mediterranean Diet Score, DASH-S= Dietary Approaches to Stop Hypertension Score, DQI-I= Diet Quality Index-International, DII= Dietary Inflammatory Index, DAI= Dietary Antioxidant Index, NNRS= Naturally Nutrient-Rich Score, SII= Systemic immune-inflammation index, BMI= Body Mass Index, WC= Waist Circumference, hsCRP= high-sensitivity C-reactive protein, HOMA-IR= Homeostatic Model Assessment for Insulin Resistance, FBS= Fasting blood sugar, TG= Triglycerides, LDL= Low-density lipoprotein, HDL= High-density lipoprotein, Mg= Magnesium, TSH= Thyroid-stimulating hormone, GFR= Glomerular Filtration Rate, CSBP= Central systolic blood pressure, CDBP= Central diastolic blood pressure, PWV= Carotid-femoral pulse wave velocity.  * E-DII (Beta= 8.550, 95%CI: 1.585-15.515; p-value= 0.016) | | | | | | | | | | | | | | | | | | | | | | | | | | | | | | | | | | | | | |
| **Supplementary Table 7.** Multivariable linear regression^a^ of the associations between diet quality indices (quartiles) and serum and metabolic biomarkers investigated in the ORISCAV-Lux-2 study. | | | | | | | | | | | | | | | | | | | | | | | | | | | | | | | | | | | | |  |
|  | AHEI | | | | | | MDS | | | | | | DASH-S | | | | | | DQI-I | | | | | DII | | | | | DAI | | | | NNRS | | | |  |
|  | β (95%CI) | | | P-value | | | β (95%CI) | | | P-value | | | β (95%CI) | | | P-value | | | β (95%CI) | | | P-value | | β (95%CI) | | | P-value | | β (95%CI) | | P-value | | β (95%CI) | | P-value | |  |
| **Anthropometry** | | | | | | | | | | | | | | | | | | | | | | | | | | | | | | | | | | | | |  |
| BMI (kg/m^2^) | -0.304 (-0.525, -0.082) | | | 0.007 | | | -0.337 (-0.551, -0.123) | | | 0.002 | | | -0.537 (-0.758, -0.316) | | | <0.001 | | | -0.374 (-0.591, -0.157) | | | 0.001 | | -0.008 (-0.231, 0.214) | | | 0.942 | | 0.451 (0.230, 0.672) | | <0.001 | | 0.458 (0.237, 0.680) | | <0.001 | |  |
| WC (cm) | -0.651 (-1.277, -0.024) | | | 0.042 | | | -1.217 (-1.822, -0.613) | | | <0.001 | | | -1.727 (-2.351, -1.103) | | | <0.001 | | | -1.256 (-1.870, -0.643) | | | <0.001 | | -0.551 (-1.181, 0.078) | | | 0.086 | | 2.067 (1.446, 2.687) | | <0.001 | | 2.114 (1.493, 2.734) | | <0.001 | |  |
| WHR | -0.005 (-0.009, -0.000) | | | 0.047 | | | -0.008 (-0.012, -0.004) | | | <0.001 | | | -0.012 (-0.017, -0.008) | | | <0.001 | | | -0.009 (-0.013, -0.004) | | | <0.001 | | -0.003 (-0.007, 0.002) | | | 0.232 | | 0.012 (0.008, 0.017) | | <0.001 | | 0.013 (0.009, 0.018) | | <0.001 | |  |
| **Inflammation related measurements** | | | | | | | | | | | | | | | | | | | | | | | | | | | | | | | | | | | | |  |
| hsCRP (μg/L) | -0.175 (-0.432, 0.081) | | | 0.180 | | | -0.145 (-0.393, 0.103) | | | 0.251 | | | -0.134 (-0.392, 0.124) | | | 0.309 | | | -0.077 (-0.330, 0.175) | | | 0.549 | | 0.176 (-0.081, 0.434) | | | 0.179 | | -0.087 (-0.344, 0.171) | | 0.509 | | -0.081 (-0.340, 0.178) | | 0.539 | |  |
| SII | -2.197 (-12.931, 8.537) | | | 0.688 | | | -6.839 (-17.194, 3.517) | | | 0.195 | | | -5.325 (-16.097, 5.446) | | | 0.332 | | | -5.996 (-16.547, 4.556) | | | 0.265 | | 0.959 (-9.813, 11.73) | | | 0.861 | | -0.584 (-11.343, 10.17) | | 0.915 | | -0.143 (-10.975, 10.69) | | 0.979 | |  |
| **Glucose related measurements** | | | | | | | | | | | | | | | | | | | | | | | | | | | | | | | | | | | | |  |
| Insulin (μg/L) | -0.575 (-1.147, -0.002) | | | 0.049 | | | -0.406 (-0.959, 0.147) | | | 0.150 | | | 0.305 (-0.270, 0.880) | | | 0.298 | | | -0.075 (-0.639, 0.488) | | | 0.793 | | 0.446 (-0.128, 1.020) | | | 0.128 | | 0.139 (-0.435, 0.713) | | 0.635 | | 0.147 (-0.431, 0.724) | | 0.619 | |  |
| HOMA-IR | -4.571 (-9.026, -0.116) | | | 0.044 | | | -3.366 (-7.664, 0.932) | | | 0.125 | | | 2.752 (-1.729, 7.233) | | | 0.228 | | | -0.717 (-5.109, 3.676) | | | 0.749 | | 3.837 (-0.633, 8.307) | | | 0.092 | | 0.417 (-4.053, 4.886) | | 0.855 | | 0.198 (-4.304, 4.701) | | 0.931 | |  |
| HbA1c (%) | 0.148 (-0.106, 0.402) | | | 0.253 | | | 0.013 (-0.232, 0.257) | | | 0.919 | | | 0.401 (0.148, 0.655) | | | 0.002 | | | 0.291 (0.042, 0.540) | | | 0.022 | | -0.019 (-0.274, 0.236) | | | 0.884 | | 0.220 (-0.034, 0.474) | | 0.090 | | 0.095 (-0.161, 0.350) | | 0.649 | |  |
| FBS (mg/dL) | -0.508 (-1.330, 0.314) | | | 0.226 | | | -1.051 (-1.842, -0.259) | | | 0.009 | | | -0.577 (-1.401, 0.247) | | | 0.170 | | | -0.462 (-1.270, 0.346) | | | 0.262 | | 0.091 (-0.734, 0.916) | | | 0.829 | | 1.088 (0.266, 1.910) | | 0.010 | | 0.834 (0.006, 1.661) | | 0.048 | |  |
| **Lipid related measurements** | | | | | | | | | | | | | | | | | | | | | | | | | | | | | | | | | | | | |  |
| Apo A (mg/L) | -0.895 (-2.238, 0.448) | | | 0.191 | | | -0.850 (-2.148, 0.449) | | | 0.199 | | | 1.783 (0.435, 3.130) | | | 0.010 | | | -0.590 (-1.912, 0.732) | | | 0.382 | | 1.428 (0.082, 2.773) | | | 0.038 | | -3.060 (-4.398, -1.721) | | <0.001 | | -3.314 (-4.658, -1.970) | | <0.001 | |  |
| Apo B (mg/L) | -0.546 (-1.601, 0.509) | | | 0.310 | | | -0.691 (-1.711, 0.329) | | | 0.184 | | | -1.888 (-2.944, -0.831) | | | <0.001 | | | -1.497 (-2.533, -0.461) | | | 0.005 | | -0.646 (-1.704, 0.412) | | | 0.231 | | 1.337 (0.280, 2.394) | | 0.013 | | 1.565 (0.504, 2.627) | | 0.004 | |  |
| TG (mg/dL) | -6.775 (-11.12, -2.434) | | | 0.002 | | | -4.952 (-9.147, -0.757) | | | 0.021 | | | -8.798 (-13.143, -4.454) | | | <0.001 | | | -5.761 (-10.028, -1.493) | | | 0.008 | | 4.305 (-0.054, 8.665) | | | 0.053 | | 1.629 (-2.738, 5.996) | | 0.464 | | 2.715 (-1.669, 7.099) | | 0.225 | |  |
| Total cholesterol (mg/dL) | -0.563 (-2.371, 1.245) | | | 0.541 | | | -0.723 (-2.468, 1.022) | | | 0.416 | | | -0.955 (-2.768, 0.859) | | | 0.302 | | | -1.748 (-3.522, 0.026) | | | 0.053 | | -0.915 (-2.727, 0.897) | | | 0.322 | | 0.460 (-1.354, 2.273) | | 0.619 | | 0.505 (-1.316, 2.326) | | 0.587 | |  |
| LDL (mg/dL) | 0.298 (-1.299, 1.894) | | | 0.714 | | | -0.042 (-1.582, 1.498) | | | 0.957 | | | -1.334 (-2.934, 0.267) | | | 0.102 | | | -1.144 (-2.711, 0.422) | | | 0.152 | | -1.539 (-3.137, 0.059) | | | 0.059 | | 1.499 (-0.100, 3.099) | | 0.066 | | 1.536 (-0.070, 3.142) | | 0.061 | |  |
| HDL (mg/dL) | 0.338 (-0.347, 1.023) | | | 0.333 | | | 0.377 (-0.284, 1.038) | | | 0.263 | | | 1.818 (1.137, 2.498) | | | <0.001 | | | 0.544 (-0.128, 1.216) | | | 0.113 | | 0.008 (-0.679, 0.695) | | | 0.981 | | -1.498 (-2.181, -0.815) | | <0.001 | | -1.697 (-2.381, -1.012) | | <0.001 | |  |
| **Kidney related measurement** | | | | | | | | | | | | | | | | | | | | | | | | | | | | | | | | | | | | |  |
| Urinary microalbumin (mg/L) | -1.276 (-4.008, 1.456) | | | 0.360 | | | -0.454 (-3.084, 2.175) | | | 0.735 | | | -0.507 (-3.251, 2.236) | | | 0.717 | | | -1.069 (-3.751, 1.613) | | | 0.434 | | 2.199 (-0.536, 4.935) | | | 0.115 | | -1.065 (-3.811, 1.681) | | 0.447 | | -1.834 (-4.597, 0.928) | | 0.193 | |  |
| Urinary creatinine (μM) | -11.985 (-15.58, -8.39) | | | <0.001 | | | -10.154 (-13.62, -6.68) | | | <0.001 | | | -13.325 (-16.922, -9.73) | | | <0.001 | | | -11.848 (-15.377, -8.32) | | | <0.001 | | 3.438 (-0.220, 7.095) | | | 0.065 | | -0.055 (-3.729, 3.619) | | 0.977 | | 2.488 (-1.207, 6.183) | | 0.187 | |  |
| Albumin/creatinine | -0.902 (-4.623, 2.820) | | | 0.635 | | | -0.911 (-4.462, 2.641) | | | 0.615 | | | -0.521 (-4.257, 3.214) | | | 0.784 | | | -0.724 (-4.409, 2.962) | | | 0.700 | | 2.813 (-0.950, 6.576) | | | 0.143 | | -2.363 (-6.073, 1.348) | | 0.212 | | -2.312 (-6.049, 1.424) | | 0.225 | |  |
| Uric acid (mg/dL) | -0.092 (-0.154, -0.030) | | | 0.004 | | | -0.130 (-0.190, -0.070) | | | <0.001 | | | -0.233 (-0.294, -0.172) | | | <0.001 | | | -0.141 (-0.201, -0.080) | | | <0.001 | | -0.047 (-0.109, 0.015) | | | 0.138 | | 0.125 (0.064, 0.187) | | <0.001 | | 0.153 (0.090, 0.215) | | <0.001 | |  |
| **Nutritional status** | | | | | | | | | | | | | | | | | | | | | | | | | | | | | | | | | | | | |  |
| Plasma Vitamin D (ng/mL) | 1.326 (0.801, 1.852) | | | <0.001 | | | 0.817 (0.307, 1.328) | | | 0.002 | | | 1.390 (0.863, 1.918) | | | <0.001 | | | 1.050 (0.531, 1.568) | | | <0.001 | | -0.633 (-1.16, -0.103) | | | 0.019 | | 0.001 (-0.531, 0.533) | | 0.997 | | -0.219 (-0.754, 0.315) | | 0.421 | |  |
| Calcium in serum (mg/dL) | -0.011 (-0.028, 0.007) | | | 0.230 | | | -0.004 (-0.020, 0.013) | | | 0.667 | | | -0.019 (-0.036, -0.002) | | | 0.032 | | | -0.005 (-0.022, 0.012) | | | 0.956 | | -0.003 (-0.020, 0.014) | | | 0.732 | | 0.006 (-0.012, 0.023) | | 0.518 | | 0.008 (-0.010, 0.025) | | 0.394 | |  |
| Urinary sodium (mg/dL) | -2.379 (-4.538, -0.220) | | | 0.031 | | | -1.530 (-3.690, 0.548) | | | 0.149 | | | -4.883 (-7.037, -2.729) | | | <0.001 | | | -3.807 (-5.918, -1.695) | | | <0.001 | | 0.854 (-1.313, 3.020) | | | 0.440 | | 2.160 (-0.011, 4.331) | | 0.051 | | 3.055 (0.874, 5.237) | | 0.006 | |  |
| Sodium in serum (mg/dL) | 0.083 (-0.002, 0.168) | | | 0.056 | | | 0.053 (-0.029, 0.135) | | | 0.204 | | | 0.011 (-0.074, 0.097) | | | 0.794 | | | 0.053 (-0.031, 0.136) | | | 0.214 | | -0.075 (-0.160, 0.010) | | | 0.084 | | 0.095 (0.009, 0.180) | | 0.029 | | 0.103 (0.018, 0.189) | | 0.018 | |  |
| Potassium serum (mg/dL) | -0.005 (-0.019, 0.010) | | | 0.530 | | | -0.004 (-0.018, 0.009) | | | 0.528 | | | -0.007 (-0.021, 0.008) | | | 0.369 | | | -0.005 (-0.019, 0.009) | | | 0.457 | | -0.001 (-0.015, 0.013) | | | 0.888 | | 0.001 (-0.013, 0.015) | | 0.911 | | 0.002 (-0.013, 0.016) | | 0.809 | |  |
| Mg in serum (mg/dL) | 0.003 (-0.003, 0.010) | | | 0.345 | | | -0.001 (-0.007, 0.006) | | | 0.859 | | | 0.001 (-0.006, 0.008) | | | 0.717 | | | 0.003 (-0.004, 0.010) | | | 0.373 | | -0.004 (-0.010, 0.003) | | | 0.275 | | -0.001 (-0.008, 0.005) | | 0.675 | | -0.001 (-0.008, 0.006) | | 0.744 | |  |
| Ferritin (ng/mL) | -7.595 (-14.13, -1.061) | | | 0.023 | | | -9.934 (-16.24, -3.627) | | | 0.002 | | | -19.705 (-26.21, -13.21) | | | <0.001 | | | -11.940 (-18.351, -5.53) | | | <0.001 | | -2.566 (-9.126, 3.994) | | | 0.443 | | 10.808 (4.269, 17.348) | | 0.001 | | 13.865 (7.302, 20.428) | | <0.001 | |  |
| Haematocrit (%) | -0.150 (-0.316, 0.017) | | | 0.078 | | | -0.370 (-0.530, -0.210) | | | <0.001 | | | -0.507 (-0.672, -0.342) | | | <0.001 | | | -0.350 (-0.514, 0.187) | | | <0.001 | | -0.177 (-0.344, -0.010) | | | 0.038 | | 0.434 (0.269, 0.600) | | <0.001 | | 0.491 (0.324, 0.657) | | <0.001 | |  |
| Hemoglobin (g/L) | -0.067 (-0.127, -0.006) | | | 0.032 | | | -0.151 (-0.209, -0.093) | | | <0.001 | | | -0.195 (-0.256, -0.135) | | | <0.001 | | | -0.141 (-0.201, -0.082) | | | <0.001 | | -0.060 (-0.121, 0.001) | | | 0.052 | | 0.165 (0.105, 0.225) | | <0.001 | | 0.185 (0.125, 0.246) | | <0.001 | |  |
| **Hormonal status** | | | | | | | | | | | | | | | | | | | | | | | | | | | | | | | | | | | | |  |
| TSH (mIU/L) | -0.057 (-0.108, -0.007) | | | 0.025 | | | -0.046 (-0.095, 0.002) | | | 0.062 | | | -0.045 (-0.096, 0.005) | | | 0.081 | | | -0.048 (-0.097, 0.002) | | | 0.060 | | 0.039 (-0.012, 0.089) | | | 0.132 | | -0.029 (-0.079, 0.022) | | 0.266 | | -0.007 (-0.058, 0.044) | | 0.788 | |  |
| Free T3 (pmol/L) | -0.014 (-0.034, 0.007) | | | 0.186 | | | -0.016 (-0.035, 0.004) | | | 0.117 | | | -0.012 (-0.032, 0.008) | | | 0.245 | | | -0.005 (-0.025, 0.015) | | | 0.611 | | -0.003 (-0.023, 0.018) | | | 0.786 | | 0.007 (-0.014, 0.027) | | 0.512 | | 0.003 (-0.018, 0.023) | | 0.790 | |  |
| Free T4 (ng/dL) | 0.007 (-0.009, 0.023) | | | 0.388 | | | 0.004 (-0.011, 0.020) | | | 0.585 | | | 0.003 (-0.013, 0.019) | | | 0.741 | | | 0.002 (-0.013, 0.018) | | | 0.779 | | -0.008 (-0.024, 0.008) | | | 0.348 | | 0.009 (-0.007, 0.025) | | 0.253 | | 0.008 (-0.008, 0.024) | | 0.316 | |  |
| **Vascular function and cardiovascular risk** | | | | | | | | | | | | | | | | | | | | | | | | | | | | | | | | | | | | |  |
| SBP (mmHg) | -0.496 (-1.284, 0.293) | | | 0.218 | | | -2.018 (-2.774, -1.262) | | | <0.001 | | | -1.124 (-1.913, -0.334) | | | 0.005 | | | -0.972 (-1.746, -0.198) | | | 0.014 | | -0.135 (-0.926, 0.657) | | | 0.738 | | 0.735 (-0.055, 1.525) | | 0.068 | | 0.620 (-0.171, 1.411) | | 0.125 | |  |
| CSBP (mmHg) | -0.824 (-1.632, -0.016) | | | 0.046 | | | -2.225 (-3.003, -1.447) | | | <0.001 | | | -1.486 (-2.298, -0.673) | | | <0.001 | | | -1.124 (-1.922, -0.326) | | | 0.006 | | 0.110 (-0.706, 0.925) | | | 0.792 | | 0.421 (-0.396, 1.237) | | 0.312 | | 0.369 (-0.451, 1.189) | | 0.377 | |  |
| DBP (mmHg) | -0.791 (-1.287, -0.295) | | | 0.002 | | | -1.243 (-1.721, -0.766) | | | <0.001 | | | -1.052 (-1.548, -0.555) | | | <0.001 | | | -0.779 (-1.266, -0.291) | | | 0.002 | | 0.203 (-0.296, 0.702) | | | 0.425 | | 0.264 (-0.235, 0.762) | | 0.300 | | 0.134 (-0.366, 0.634) | | 0.599 | |  |
| CDBP (mmHg) | -0.939 (-1.397, -0.482) | | | <0.001 | | | -1.207 (-1.649, -0.764) | | | <0.001 | | | -0.919 (-1.380, -0.457) | | | <0.001 | | | -0.742 (-1.195, -0.289) | | | 0.001 | | 0.369 (-0.094, 0.832) | | | 0.118 | | 0.088 (-0.376, 0.552) | | 0.710 | | -0.030 (-0.496, 0.436) | | 0.901 | |  |
| GFR^b^ (ml/min/1.73m^2^) | 0.146 (-0.468, 0.760) | | | 0.641 | | | -0.107 (-0.700, 0.486) | | | 0.724 | | | -0.712 (-1.328, -0.096) | | | 0.024 | | | -0.391 (-0.995, 0.212) | | | 0.204 | | -0.555 (-1.170, 0.060) | | | 0.077 | | 0.766 (0.152, 1.381) | | 0.015 | | 0.864 (0.247, 1.482) | | 0.006 | |  |
| PWV (m/s) | 0.028 (-0.285, 0.341) | | | 0.859 | | | 0.314 (0.010, 0.618) | | | 0.043 | | | 0.205 (-0.111, 0.520) | | | 0.203 | | | 0.239 (-0.070, 0.548) | | | 0.130 | | 0.148 (-0.168, 0.463) | | | 0.358 | | -0.203 (-0.518, 0.113) | | 0.208 | | -0.259 (-0.576, 0.057) | | 0.108 | |  |
| Vascular age (y) | -1.098 (-1.981, -0.216) | | | 0.015 | | | -0.878 (-1.742, -0.013) | | | 0.047 | | | -0.639 (-1.529, 0.251) | | | 0.159 | | | -0.589 (-1.461, 0.283) | | | 0.185 | | 0.976 (0.086, 1.866) | | | 0.032 | | -0.536 (-1.428, 0.355) | | 0.238 | | -0.704 (-1.598, 0.191) | | 0.123 | |  |
| ^a^Crude models; ^b^Estimate by Modification of Diet in Renal Disease (MDRD) method.  AHEI= Alternative Healthy Eating Index, MDS= Mediterranean Diet Score, DASH-S= Dietary Approaches to Stop Hypertension Score, DQI-I= Diet Quality Index-International, DII= Dietary Inflammatory Index, DAI= Dietary Antioxidant Index, NNRS= Naturally Nutrient-Rich Score, SII= Systemic immune-inflammation index, BMI= Body Mass Index, WC= Waist Circumference, hsCRP= high-sensitivity C-reactive protein, HOMA-IR= Homeostatic Model Assessment for Insulin Resistance, FBS= Fasting blood sugar, TG= Triglycerides, LDL= Low-density lipoprotein, HDL= High-density lipoprotein, Mg= Magnesium, TSH= Thyroid-stimulating hormone, GFR= Glomerular Filtration Rate, CSBP= Central systolic blood pressure, CDBP= Central diasystolic blood pressure, PWV= Carotid-femoral pulse wave velocity. | | | | | | | | | | | | | | | | | | | | | | | | | | | | | | | | | | | | |  |
| **Supplementary Table 8.** Multivariable linear regression^a^ of the associations between diet quality indices (quartiles) and serum and metabolic biomarkers investigated in the ORISCAV-LUX-2 study. | | | | | | | | | | | | | | | | | | | | | | | | | | | | | | | | | | | | | |
|  | | AHEI | | | | | | | MDS | | | | | | DASH-S | | | | | | DQI-I | | | | | DII | | | | DAI | | | | NNRS | | | |
|  |  | β (95%CI) | | | | P-value | | | β (95%CI) | | | P-value | | | β (95%CI) | | | P-value | | | β (95%CI) | | P-value | | | β (95%CI) | | P-value | | β (95%CI) | | P-value | | β (95%CI) | | P-value | |
| **Anthropometry** | | | | | | | | | | | | | | | | | | | | | | | | | | | | | | | | | | | | | |
| BMI (kg/m^2^) | | -0.398 (-0.664, -0.133) | | | | 0.003 | | | -0.398 (-0.647, -0.130) | | | 0.003 | | | -0.481 (-0.754, -0.208) | | | 0.001 | | | -0.461 (-0.721, -0.201) | | 0.001 | | | 0.099 (-0.170, 0.368) | | 0.469 | | 0.272 (0.000, 0.545) | | 0.050 | | 0.326 (0.052, 0.599) | | 0.020 | |
| WC (cm) | | -0.866 (-1.535, -0.197) | | | | 0.011 | | | -0.997 (-1.648, -0.346) | | | 0.003 | | | -1.162 (-1.849, -0.475) | | | 0.001 | | | -1.181 (-1.835, -0.527) | | <0.001 | | | -0.048 (-0.726, 0.629) | | 0.888 | | 1.107 (0.425, 1.789) | | 0.002 | | 1.217 (0.532, 1.903) | | 0.001 | |
| WHR | | -0.005 (-0.009, -0.001) | | | | 0.025 | | | -0.003 (-0.007, 0.001) | | | 0.153 | | | -0.005 (-0.009, -0.001) | | | 0.023 | | | -0.004 (-0.007, 0.000) | | 0.077 | | | 0.001 (-0.003, 0.005) | | 0.542 | | 0.003 (-0.001, 0.007) | | 0.179 | | 0.003 (-0.001, 0.008) | | 0.107 | |
| **Inflammation related measurements** | | | | | | | | | | | | | | | | | | | | | | | | | | | | | | | | | | | | | |
| hsCRP (μg/L) | | 0.096 (-0.130, 0.323) | | | | 0.404 | | | -0.017 (-0.238, 0.204) | | | 0.879 | | | -0.087 (-0.321, 0.147) | | | 0.465 | | | 0.087 (-0.135, 0.309) | | 0.443 | | | -0.167 (-0.394, 0.061) | | 0.151 | | 0.290 (0.059, 0.520) | | 0.014 | | 0.245 (0.012, 0.478) | | 0.039 | |
| SII | | -4.446 (-18.700, 9.808) | | | | 0.541 | | | -8.032 (-21.893, 5.828) | | | 0.256 | | | -9.019 (-23.713, 5.676) | | | 0.229 | | | -6.546 (-20.521, 7.429) | | 0.358 | | | 6.667 (-7.672, 21.006) | | 0.362 | | 0.345 (-14.225, 14.92) | | 0.963 | | -0.782 (-15.521, 13.96) | | 0.917 | |
| **Glucose related measurements** | | | | | | | | | | | | | | | | | | | | | | | | | | | | | | | | | | | | | |
| Insulin (μg/L) | | -0.478 (-1.134, 0.177) | | | | 0.152 | | | -0.321 (-0.958, 0.316) | | | 0.323 | | | 0.141 (-0.534, 0.815) | | | 0.683 | | | -0.034 (-0.676, 0.603) | | 0.918 | | | 0.185 (-0.474, 0.844) | | 0.582 | | 0.215 (-0.455, 0.884) | | 0.529 | | 0.234 (-0.441, 0.909) | | 0.497 | |
| HOMA-IR | | -2.099 (-5.522, 1.324) | | | | 0.229 | | | -1.567 (-4.893, 1.758) | | | 0.355 | | | 0.977 (-2.560, 4.513) | | | 0.588 | | | 0.055 (-3.310, 3.419) | | 0.975 | | | 0.621 (-2.828, 4.070) | | 0.724 | | 1.209 (-2.298, 4.716) | | 0.499 | | 1.217 (-2.328, 4.761) | | 0.501 | |
| HbA1c (%) | | 0.218 (-0.030, 0.465) | | | | 0.084 | | | 0.025 (-0.216, 0.266) | | | 0.839 | | | 0.201 (-0.053, 0.456) | | | 0.120 | | | 0.231 (-0.011, 0.473) | | 0.061 | | | -0.222 (-0.471, 0.027) | | 0.081 | | 0.277 (0.025, 0.530) | | 0.031 | | 0.177 (-0.079, 0.433) | | 0.175 | |
| FBS (mg/dL) | | 0.066 (-0.630, 0.762) | | | | 0.853 | | | -0.510 (-1.185, 0.165) | | | 0.138 | | | -0.315 (-1.029, 0.399) | | | 0.387 | | | -0.027 (-0.708, 0.653) | | 0.937 | | | -0.559 (-1.258, 0.140) | | 0.117 | | 0.717 (0.009, 1.426) | | 0.047 | | 0.485 (-0.232, 1.202) | | 0.184 | |
| **Lipid related measurements** | | | | | | | | | | | | | | | | | | | | | | | | | | | | | | | | | | | | | |
| Apo A (mg/L) | | -1.538 (-3.048, -0.027) | | | | 0.046 | | | -1.825 (-3.295, -0.355) | | | 0.015 | | | -1.074 (-2.636, 0.487) | | | 0.177 | | | -2.481 (-3.985, -1.005) | | 0.001 | | | 0.822 (-0.698, 2.343) | | 0.289 | | -1.422 ( -2.966, 0.121) | | 0.071 | | -1.635 (-3.193, -0.077) | | 0.040 | |
| Apo B (mg/L) | | -0.503 (-1.805, 0.798) | | | | 0.448 | | | -0.186 (-1.455, 1.082) | | | 0.733 | | | -1.478 (-2.819, -0.137) | | | 0.031 | | | -1.300 (-2.574, -0.025) | | 0.046 | | | -0.530 (-1.873, 0.778) | | 0.427 | | 1.122 (-0.206, 2.449) | | 0.098 | | 1.123 (-0.219, 2.464) | | 0.101 | |
| TG (mg/dL) | | -7.089 (-13.185, -0.993) | | | | 0.023 | | | -3.202 (-9.140, 2.737) | | | 0.290 | | | -7.213 (-13.481, -0.946) | | | 0.024 | | | -2.212 (-8.192, 3.767) | | 0.468 | | | 6.480 (0.354, 12.606) | | 0.038 | | -2.956 (-9.193, 3.280) | | 0.352 | | -2.287 (-8.580, 4.007) | | 0.476 | |
| Total cholesterol (mg/dL) | | -1.072 (-3.333, 1.190) | | | | 0.353 | | | -0.428 (-2.627, 1.771) | | | 0.703 | | | -1.894 (-4.218, 0.429) | | | 0.110 | | | -2.429 (-4.637, -0.222) | | 0.031 | | | -0.828 (-3.100, 1.444) | | 0.475 | | 1.081 (-1.227, 3.389) | | 0.358 | | 0.924 (-1.405, 3.253) | | 0.437 | |
| LDL (mg/dL) | | 0.026 (-1.972, 2.023) | | | | 0.980 | | | 0.462 (-1.477, 2.401) | | | 0.640 | | | -1.333 (-3.384, 0.718) | | | 0.202 | | | -1.455 (-3.405, 0.494) | | 0.143 | | | -1.425 (-3.429, 0.579) | | 0.163 | | 1.904 (-0.131, 3.939) | | 0.067 | | 1.689 (-0.365, 3.743) | | 0.107 | |
| HDL (mg/dL) | | 0.054 (-0.691, 0.800) | | | | 0.887 | | | -0.082 (-0.805, 0.642) | | | 0.825 | | | 0.472 (-0.294, 1.237) | | | 0.227 | | | -0.376 (-1.104, 0.353) | | 0.312 | | | -0.273 (-1.022, 0.476) | | 0.475 | | -0.585 (-1.345, 0.175) | | 0.131 | | -0.659 (-1.426, 0.107) | | 0.092 | |
| **Kidney related measurement** | | | | | | | | | | | | | | | | | | | | | | | | | | | | | | | | | | | | | |
| Urinary microalbumin (mg) | | -1.342 (-5.347, 2.663) | | | | 0.511 | | | -0.223 (-4.137, 3.692) | | | 0.911 | | | 0.164 (-3.983, 4.311) | | | 0.938 | | | -0.767 (-4.718, 3.184) | | 0.703 | | | 3.341 (-0.676, 7.357) | | 0.103 | | -1.559 (-5.649, 2.531) | | 0.455 | | -2.573 (-6.721, 1.575) | | 0.224 | |
| Urinary creatinine (μmol) | | -10.648 (-14.875, -6.422) | | | | <0.001 | | | -5.960 (-10.131, -1.789) | | | 0.005 | | | -6.275 (-10.694, -1.856) | | | 0.005 | | | -5.340 (-9.554, -1.126) | | 0.013 | | | 6.310 (2.025, 10.594) | | 0.004 | | -6.049 (-10.409, -1.69) | | 0.007 | | -3.992 (-8.428, 0.445) | | 0.078 | |
| Albumin/creatinine | | -1.883 (-7.586, 3.819) | | | | 0.517 | | | -2.392 (-7.936, 3.153) | | | 0.397 | | | -2.402 (-8.302, 3.499) | | | 0.424 | | | -2.182 (-7.923, 3.559) | | 0.456 | | | 3.709 (-2.110, 9.528) | | 0.211 | | -2.639 (-8.423, 3.146) | | 0.371 | | -2.170 (-8.074, 3.734) | | 0.470 | |
| Uric acid (mg/dL) | | -0.079 (-0.144, -0.014) | | | | 0.018 | | | -0.069 (-0.133, -0.006) | | | 0.033 | | | -0.144 (-0.211, -0.078) | | | <0.001 | | | -0.081 (-0.145, -0.017) | | 0.013 | | | 0.005 (-0.061, 0.070) | | 0.891 | | 0.008 (-0.059, 0.075) | | 0.816 | | 0.009 (-0.058, 0.077) | | 0.786 | |
| **Nutritional status** | | | | | | | | | | | | | | | | | | | | | | | | | | | | | | | | | | | | | |
| Plasma Vitamin D (ng/mL) | | 1.493 (0.849, 2.136) | | | | <0.001 | | | 0.527 (-0.107, 1.161) | | | 0.103 | | | 0.960 (0.290, 1.629) | | | 0.005 | | | 0.765 (0.128, 1.402) | | 0.019 | | | -1.040 (-1.691, -0.389) | | 0.002 | | 0.838 (0.176, 1.501) | | 0.013 | | 0.531 (-0.140, 1.202) | | 0.121 | |
| Calcium in serum (mg/dL) | | 0.006 (-0.017, 0.028) | | | | 0.620 | | | 0.010 (-0.013, 0.032) | | | 0.396 | | | 0.001 (-0.022, 0.025) | | | 0.927 | | | 0.013 (-0.009, 0.035) | | 0.258 | | | -0.001 (-0.024, 0.022) | | 0.926 | | -0.005 (-0.028, 0.018) | | 0.656 | | -0.008 (-0.032, 0.015) | | 0.497 | |
| Urinary sodium (mg/dL) | | -1.733 (-4.356, 0.889) | | | | 0.195 | | | -1.305 (-3.867, 1.257) | | | 0.318 | | | -4.438 (-7.137, -1.739) | | | 0.001 | | | -3.924 (-6.498, -1.351) | | 0.003 | | | 1.331 (-1.305, 3.966) | | 0.322 | | 2.032 (-0.647, 4.712) | | 0.137 | | 3.552 (0.840, 6.264) | | 0.010 | |
| Sodium in serum (mg/dL) | | 0.077 (-0.031, 0.185) | | | | 0.162 | | | 0.059 (-0.046, 0.165) | | | 0.271 | | | -0.012 (-0.123, 0.100) | | | 0.838 | | | 0.043 (-0.063, 0.149) | | 0.425 | | | -0.075 (-0.183, 0.034) | | 0.178 | | 0.115 (0.004, 0.225) | | 0.041 | | 0.134 (0.023, 0.246) | | 0.018 | |
| Potassium serum (mg/dL) | | -0.004 (-0.021, 0.014) | | | | 0.686 | | | -0.007 (-0.024, 0.010) | | | 0.423 | | | -0.012 (-0.030, 0.007) | | | 0.206 | | | -0.006 (-0.024, 0.011) | | 0.492 | | | -0.003 (-0.021, 0.015) | | 0.774 | | 0.008 (-0.011, 0.026) | | 0.413 | | 0.012 (-0.007, 0.030) | | 0.215 | |
| Mg in serum (mg/dL) | | 0.004 (-0.005, 0.013) | | | | 0.353 | | | 0.003 (-0.005, 0.012) | | | 0.435 | | | 0.002 (-0.007, 0.011) | | | 0.613 | | | 0.007 (-0.002, 0.016) | | 0.109 | | | -0.001 (-0.010, 0.008) | | 0.824 | | -0.006 (-0.015, 0.003) | | 0.211 | | -0.003 (-0.012, 0.006) | | 0.515 | |
| Ferritin (ng/L) | | -1.706 (-9.058, 5.646) | | | | 0.649 | | | 0.495 (-6.668, 7.658) | | | 0.892 | | | -6.099 (-13.680, 1.482) | | | 0.115 | | | -0.875 (-8.088, 6.339) | | 0.812 | | | -0.882 (-8.269, 6.506) | | 0.815 | | 0.382 (-7.128, 7.891) | | 0.921 | | 3.344 (-4.237, 10.925) | | 0.387 | |
| Haematocrit (%) | | -0.009 (-0.166, 0.147) | | | | 0.909 | | | -0.042 (-0.194, 0.110) | | | 0.588 | | | -0.068 (-0.230, 0.093) | | | 0.406 | | | -0.011 (-0.164, 0.143) | | 0.892 | | | -0.020 (-0.178, 0.138) | | 0.802 | | 0.038 (-0.122, 0.199) | | 0.638 | | 0.079 (-0.083, 0.241) | | 0.339 | |
| Hemoglobin (g/L) | | -0.016 (-0.069, 0.037) | | | | 0.556 | | | -0.030 (-0.082, 0.022) | | | 0.255 | | | -0.027 (-0.081, 0.028) | | | 0.341 | | | -0.010 (-0.062, 0.043) | | 0.717 | | | -0.002 (-0.056, 0.051) | | 0.934 | | 0.015 (-0.040, 0.069) | | 0.599 | | 0.027 (-0.028, 0.082) | | 0.338 | |
| **Hormonal status** | | | | | | | | | | | | | | | | | | | | | | | | | | | | | | | | | | | | | |
| TSH (mIU/L) | | -0.082 (-0.146, -0.019) | | | | 0.011 | | | -0.069 (-0.131, -0.007) | | | 0.028 | | | -0.061 (-0.127, 0.005) | | | 0.070 | | | -0.070 (-0.132, -0.008) | | 0.028 | | | 0.060 (-0.004, 0.124) | | 0.067 | | -0.030 (-0.095, 0.035) | | 0.365 | | -0.007 (-0.072, 0.059) | | 0.845 | |
| Free T3 (pmol/L) | | -0.009 (-0.035, 0.018) | | | | 0.524 | | | -0.010 (-0.036, 0.016) | | | 0.464 | | | 0.007 (-0.020, 0.035) | | | 0.604 | | | 0.008 (-0.019, 0.034) | | 0.570 | | | 0.003 (-0.024, 0.030) | | 0.805 | | -0.003 (-0.030, 0.025) | | 0.853 | | -0.016 (-0.044, 0.011) | | 0.250 | |
| Free T4 (ng/dL) | | 0.013 (-0.011, 0.038) | | | | 0.290 | | | 0.002 (-0.022, 0.026) | | | 0.873 | | | -0.000 (-0.025. 0.026) | | | 0.969 | | | 0.001 (0.023, 0.025) | | 0.933 | | | -0.014 (-0.039, 0.010) | | 0.255 | | 0.019 (-0.006, 0.044) | | 0.140 | | 0.018 (-0.008, 0.043) | | 0.171 | |
| **Vascular function and cardiovascular risk** | | | | | | | | | | | | | | | | | | | | | | | | | | | | | | | | | | | | | |
| SBP (mmHg) | | -0.866 (-1.726, -0.006) | | | | 0.048 | | | -1.663 (-2.494, -0.831) | | | <0.001 | | | -1.228 (-2.110, -0.3459 | | | 0.006 | | | -1.127 (-1.968, -0.285) | | 0.009 | | | 0.389 (-0.479, 1.256) | | 0.379 | | -0.025 (-0.905, 0.855) | | 0.956 | | -0.272 (-1.157, 0.613) | | 0.546 | |
| CSBP (mmHg) | | -0.811 (-1.689, 0.067) | | | | 0.070 | | | -1.754 (-2.605, -0.903) | | | <0.001 | | | -1.327 (-2.235, -0.419) | | | 0.004 | | | -1.352 (-2.214, -0.490) | | 0.002 | | | 0.484 (-0.402, 1.370) | | 0.284 | | -0.151 (-1.052, 0.749) | | 0.742 | | -0.230 (-1.136, 0.677) | | 0.619 | |
| DBP (mmHg) | | -0.892 (-1.490, -0.293) | | | | 0.004 | | | -1.020 (-1.601, -0.438) | | | 0.001 | | | -0.839 (-1.455, -0.223) | | | 0.008 | | | -0.536 (-1.125, 0.052) | | 0.074 | | | 0.459 (-0.146, 1.064) | | 0.137 | | -0.206 (-0.820, 0.408) | | 0.510 | | -0.453 (-1.070, 0.164) | | 0.150 | |
| CDBP (mmHg) | | -1.055 (-1.589, -0.521) | | | | <0.001 | | | -1.082 (-1.603, -0.561) | | | <0.001 | | | -0.820 (-1.376, -0.264) | | | 0.004 | | | -0.649 (-1.178, -0.120) | | 0.016 | | | 0.580 (0.038, 1.121) | | 0.036 | | -0.372 (-0.923, 0.179) | | 0.185 | | -0.532 (-.1086, 0.022) | | 0.060 | |
| GFR^b^ (ml/min/1.73m^2^) | | 0.171 (-0.529, 0.871) | | | | 0.632 | | | 0.091 (-0.591, 0.774) | | | 0.793 | | | 0.401 (-0.322, 1.124) | | | 0.276 | | | 0.126 (-0.562, 0.813) | | 0.720 | | | -0.198 (-0.902, 0.505) | | 0.580 | | -0.031 (-0.746, 0.684) | | 0.932 | | 0.044 (-0.678, 0.767) | | 0.905 | |
| PWV (m/s) | | -0.095 (-0.405, 0.216) | | | | 0.551 | | | 0.087 (-0.216, 0.390) | | | 0.574 | | | -0.040 (-0.363, 0.282) | | | 0.806 | | | -0.000 (-0.306, 0.306) | | 0.999 | | | 0.150 (-0.163, 0.463) | | 0.348 | | -0.075 (-0.393, 0.243) | | 0.642 | | -0.147 (-0.468, 0.173) | | 0.666 | |
| Vascular age (y) | | -1.251 (-2.241, -0.261) | | | | 0.013 | | | -0.817 (-1.794, 0.161) | | | 0.101 | | | -0.694 (-1.722, 0.333) | | | 0.185 | | | -0.259 (-1.241, 0.722) | | 0.604 | | | 0.838 (-0.162, 1.837) | | 0.100 | | -0.415 (-1.428, 0.598) | | 0.422 | | -0.708 (-1.733, 0.317) | | 0.175 | |
| ^a^Adjusted model: age (5 groups), gender, birth country, marital status, education, job, income (8 groups), IPAQ scoring, current smoking  ^b^ Estimate by Modification of Diet in Renal Disease (MDRD) method.  AHEI= Alternative Healthy Eating Index, MDS= Mediterranean Diet Score, DASH-S= Dietary Approaches to Stop Hypertension Score, DQI-I= Diet Quality Index-International, DII= Dietary Inflammatory Index, DAI= Dietary Antioxidant Index, NNRS= Naturally Nutrient-Rich Score, SII= Systemic immune-inflammation index, BMI= Body Mass Index, WC= Waist Circumference, hsCRP= high-sensitivity C-reactive protein, HOMA-IR= Homeostatic Model Assessment for Insulin Resistance, FBS= Fasting blood sugar, TG= Triglycerides, LDL= Low-density lipoprotein, HDL= High-density lipoprotein, Mg= Magnesium, TSH= Thyroid-stimulating hormone, GFR= Glomerular Filtration Rate, CSBP= Central systolic blood pressure, CDBP= Central diasystolic blood pressure, PWV= Carotid-femoral pulse wave velocity. | | | | | | | | | | | | | | | | | | | | | | | | | | | | | | | | | | | | | |

**Supplementary Table 9.** Number of significant associations between combinations of two diet quality indices and metabolic biomarkers.

| MDS (8) | 13 |  | | | | |
| --- | --- | --- | --- | --- | --- | --- |
| DASH-S (14) | 17 | 16 |  | | | |
| DQI-I (13) | 16 | 14 | 16 |  | | |
| DII (2) | 11 | 9 | 14 | 13 |  | |
| DAI (5) | 14 | 11 | 17 | 16 | 6 |  |
| NNRS (6) | 15 | 13 | 17 | 16 | 8 | 9 |
| **Indices** | AHEI (11) | MDS (8) | DASH-S (14) | DQI-I (13) | DII (2) | DAI (5) |
| Values in () are the numbers related to that index alone, and values in the table present complementary metabolic biomarkers if two indices are considered together. For example, DASH-S (14 sign. Association), if combined with DAI (5), or NNRS (6), will have the most sig. association, 17, with metabolic biomarkers.  AHEI= Alternative Healthy Eating Index, MDS= Mediterranean Diet Score, DASH-S= Dietary Approaches to Stop Hypertension Score, DQI-I= Diet Quality Index- International, DII= Dietary Inflammatory Index, DAI= Dietary Antioxidant Index, NNRS= Naturally Nutrient-Rich Score. | | | | | | |

1. McCullough ML, Feskanich D, Stampfer MJ, Giovannucci EL, Rimm EB, Hu FB, Spiegelman D, Hunter DJ, Colditz GA, Willett WC (2002) Diet quality and major chronic disease risk in men and women: moving toward improved dietary guidance. The American Journal of Clinical Nutrition 76 (6):1261-1271. doi:10.1093/ajcn/76.6.1261

2. Trichopoulou A, Kouris-Blazos A, Wahlqvist ML, Gnardellis C, Lagiou P, Polychronopoulos E, Vassilakou T, Lipworth L, Trichopoulos D (1995) Diet and overall survival in elderly people. BMJ 311 (7018):1457-1460. doi:10.1136/bmj.311.7018.1457

3. Fung TT, Chiuve SE, McCullough ML, Rexrode KM, Logroscino G, Hu FB (2008) Adherence to a DASH-Style Diet and Risk of Coronary Heart Disease and Stroke in Women. Archives of Internal Medicine 168 (7):713-720. doi:10.1001/archinte.168.7.713

4. Kim S, Haines PS, Siega-Riz AM, Popkin BM (2003) The Diet Quality Index-International (DQI-I) Provides an Effective Tool for Cross-National Comparison of Diet Quality as Illustrated by China and the United States. The Journal of Nutrition 133 (11):3476-3484. doi:10.1093/jn/133.11.3476

5. Cavicchia PP, Steck SE, Hurley TG, Hussey JR, Ma Y, Ockene IS, Hébert JR (2009) A New Dietary Inflammatory Index Predicts Interval Changes in Serum High-Sensitivity C-Reactive Protein. The Journal of Nutrition 139 (12):2365-2372. doi:10.3945/jn.109.114025

6. Wright ME, Mayne ST, Stolzenberg-Solomon RZ, Li Z, Pietinen P, Taylor PR, Virtamo J, Albanes D (2004) Development of a comprehensive dietary antioxidant index and application to lung cancer risk in a cohort of male smokers. Am J Epidemiol 160 (1):68-76. doi:10.1093/aje/kwh173

7. Drewnowski A (2005) Concept of a nutritious food: toward a nutrient density score. The American Journal of Clinical Nutrition 82 (4):721-732. doi:10.1093/ajcn/82.4.721
